# Terpenoids derived from *Semen Ziziphi Spinosae* oil enhance sleep by modulating neurotransmitter signaling in mice

**DOI:** 10.1016/j.heliyon.2024.e26979

**Published:** 2024-03-01

**Authors:** Mingzhe Sun, Mengnan Li, Xinwen Cui, Lin Yan, Yiqiao Pei, Chao Wang, Chunbo Guan, Xiuqing Zhang

**Affiliations:** a College of Food Science & Nutritional Engineering, China Agricultural University, Beijing 100083, China; b Health Foods R&D Oﬃce of Hebei Yiling Pharmaceutical Research Institute, Shijiazhuang 050035, China; c National Key Laboratory for Innovation and Transformation of Luobing Theory, Shijiazhuang 050035, China; d Key Laboratory of State Administration of TCM (Cardio-Cerebral Vessel Collateral Disease), Shijiazhuang 050035, China; e College of Food Engineering and Biotechnology, Tianjin University of Science & Technology, Tianjin 300457, China

**Keywords:** Natural products, Sleep-promoting, Terpenoids, Neurotransmitter, Anti-oxidative

## Abstract

*Semen Ziziphi Spinosae* oil (SZSO) is a natural vegetable oil extracted from *Semen Ziziphi Spinosae*, a traditional Chinese medicine renowned for its sleep-promoting properties, while the mechanisms are still unclear. Our findings revealed that the terpenoids present in SZSO (T-SZSO) were identified as the active components responsible for promoting sleep. Network pharmacological analysis suggested that T-SZSO targeted different sleep-aid pathways to varying degrees and exhibited potential for preventing central nervous system diseases. Notably, lupeol and betulinicaldehyde exhibited more pronounced effects. Additionally, T-SZSO significantly elevated serotonin levels, enhanced gamma-aminobutyric acid (GABA) synthesis, promoted GABA A receptor expression, and decreased glutamate and norepinephrine expression levels. Moreover, T-SZSO was found to downregulate IL-1β expression while upregulating superoxide dismutase and inducible nitric oxide synthase levels. In conclusion, this study presents the first investigation into the pharmacological basis of SZSO in promoting sleep and highlights the potential of nature food in improving suboptimal health conditions.

## Highlights

• Terpenoids present in SZSO are the active components for promoting sleep.
• Lupeol and betulinicaldehyde are detected in T-SZSO for the first time.
• T-SZSO modulates sleep-related neurotransmitter signaling in the mouse brain.
• T-SZSO shows neuroprotective properties by mitigating cellular oxidation and inflammation.

## Graphical abstract

**Figure:**
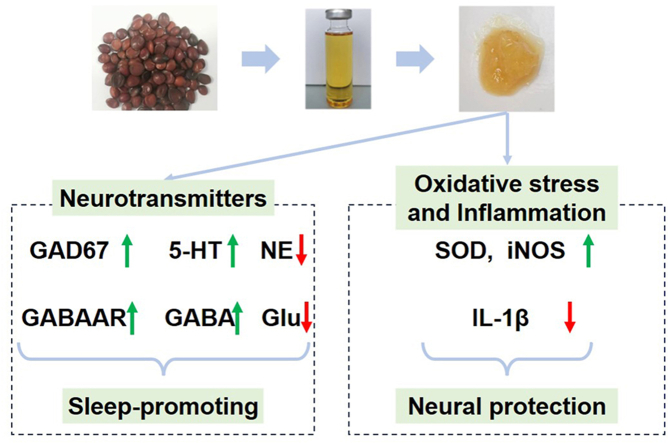

## 1 Introduction

Sleep is a complex physiological process with a significant impact on both physical and mental health. During sleep, the brain undergoes various self-repair processes that are crucial for consolidating learning and memory [1]. Additionally, deep sleep promotes hormone release to maintain tissue homeostasis, while enhancing the immune system and increasing resilience against diseases and infections [2,3]. Insomnia, characterized by difficulty falling asleep, maintaining sleep, and early awakening, can lead to a decline in central nerve and immune function, increasing the risk of various diseases such as neurodegenerative diseases, cardiovascular disease, metabolic disorders, and even cancer [[4], [5], [6]] Furthermore, insomnia can serve as a trigger for mental disorders such as anxiety and depression [7,8]. The high prevalence of insomnia in modern society imposes a significant burden on the medical system, making it an important issue that needs to be addressed. However, since improving sleep requires long-term management, medication with minimal side effects is necessary. There has been increased attention on natural functional foods and traditional Chinese herbs compared to Western medicine. The primary Chinese herbal remedies utilized for the treatment of insomnia include *Ziziphus jujuba* [9], *Schisandrae chinensis* [10], *Eucommia ulmoides* [11], *Acanthopanax Senticosus* [12], *Ganoderma* [13], and *Poria* [14].

*Semen Ziziphi Spinosae* (SZS) refers to the dried and mature seeds of *Ziziphus jujuba* Mill. var. *spinosa* (Bunge) Hu ex H. F. Chou, which possess the beneficial effects on the cardiovascular and central nerve systems [15]. SZS has a long history of being used for treating insomnia and is commonly prescribed to improve sleep quality [16]. *Semen Ziziphi Spinosae* oil (SZSO) is a mixture of lipid components, mainly composed of triglycerides and liposoluble secondary metabolites, accounting for over 30% of SZS [[17], [18], [19]] [[17], [18], [19]] [[17], [18], [19]]. Triacylglycerol are the main constituents, rich in various fatty acids (FA-SZSO) [[20], [21], [22], [23], [24], [25]] [[20], [21], [22], [23], [24], [25]] [[20], [21], [22], [23], [24], [25]]. In contrast, liposoluble secondary metabolites are relatively scarce, while terpenoids (T-SZSO) are the major metabolites [18,19]. Many studies have shown that SZSO has good effects on promoting sleep and long-term consumption does not lead to significant tolerance [[20], [21], [22], [26], [27]] [[20], [21], [22], [26], [27]] [22,26,27]. Therefore, SZSO has been widely used as a safe and effective natural plant oil to enhance sleep quality for insomnia patients. However, the limited research on the mechanism of SZSO in promoting sleep obstructs its wider application.

The regulation of sleep involves a sophisticated interplay of various factors, including circadian rhythms and neurochemical signals. Circadian genes, such as PER1 and CALM2, help synchronize the sleep-wake cycle with environmental cues such as light and darkness [28,29]. At the neuronal level, specific regions in the brain, such as the hypothalamus, brainstem, and thalamus, control the sleep-wake cycle. These regions interact with neurotransmitters like adenosine, serotonin, and gamma-aminobutyric acid (GABA) to promote or inhibit sleep [30,31]. GABA-A receptor (GABAAR) is a key target of the GABA signaling pathway. It activates Cl^−^ channels, inducing the influx of Cl^−^ ions and membrane depolarization. This inhibitory effect counteracts the arousal neurotransmitters and neuronal excitations, ultimately improving sleep quality [32,33]. Serotonin (5-HT) is another crucial inhibitory neurotransmitter in the animal brain, and its deficiency may lead to insomnia [34,35]. On the other hand, Norepinephrine (NE), a monoamine neurotransmitter, regulates wakefulness and arousal [36]. Its levels fluctuate periodically. The concentration of NE in the brain is negatively correlated with the duration of rapid eye movement sleep [37].

In the present study, we investigated the sleep-promoting effect of SZSO in mice. Chromatography-mass spectrometry analysis together with animal experiments led to the identification of T-SZSO as the primary effective components for promoting sleep. Furthermore, we employed network pharmacology analysis to explore the mechanisms underlying the sleep-promoting effect of T-SZSO and conducted subsequent validation studies at both the animal and cellular levels. These findings provide the first evidence of T-SZSO playing a crucial role in promoting sleep through neurotransmitter regulation mechanisms, and enhance our understanding of the sleep-promoting effect of SZSO by offering a more comprehensive perspective.

## 2 Materials and methods

### 2.1 Chemicals and instruments

Preparation for SZSO: The SZSO was obtained by pressing roasted SZS. After roasting, the SZS were pressed at a temperature of 80∼100 °C. The resulting oil underwent further degumming (using an oil-water-salt ratio of 50:4:0.4, stirred below 80 °C) and deacidification (using an oil-water-alkali ratio of 50:2:0.2, stirred below 75 °C). It was then dehydrated (heated to 120 °C for stirring 1.5–2 h) and subjected to decolorization treatment (heated to 100 °C after dehydration, kept for 30 min, slowly filtered during cooling to a temperature range of 60∼70 °C). The oil yield is 14.23%.

Soybean oil (COFCO, China); Potassium hydroxide, ethanol, hydrochloric acid, anhydrous sodium sulfate, methanol, sodium chloride, petroleum ether and n-hexane (Analytical Reagent grade, Tianjin Kemiou Chemical Reagent Co., Ltd, China); Boron Trifluoride-Methanol Complex (Tokyo chemical industry Co., Ltd, Japan); Iso-octane of HPLC grade (Thermo Fisher Scientific, England); Pentobarbital sodium salt (Assy 99.03%) (Merck: P11011, Scientific Research Special); Fatty Acid Methyl Ester (FAME) (Sigma, America); Diazepam was purchased from Hebei Provincial Hospital of Traditional Chinese Medicine, China.

Gas chromatograph (Equipped with FID detector), Nexis GC-2030, Shimadzu Technology Co., Ltd., Japan. Gas chromatography-mass spectrometry instrument, Agilent 7890B/5977B, Agilent Technologies Inc., America. Small animal activity recorder, ZH-YLS-1c, Beijing Haifuda Technology Co., Ltd., China. pH meter, FE20K, Mettler Toledo, Switzerland. Tissue precision homogenizer, PRO200, Pro Scientific, America.

### 2.2 Animal maintenance

Seventy SPF male ICR mice weighing between 18 and 22 g was obtained from Beijing HFK bioscience CO., Ltd. All animals were maintained under a specific-pathogen-free (SPF) condition room with a 12-h light/dark cycle. They were housed at ambient air temperature (23 ± 2 °C) under humidity (55%) and fed ad libitum with distilled water and rodent chow. The animal experiments were carried out according to the ethical guidelines of the Ethics Committee of Hebei Yiling Pharmaceutical Research Institute (approval number: N2022089).

### 2.3 Sleep test

For sleep test of SZSO, twenty mice were randomly allocated into four groups: a control group (treated with soybean oil), a Diazepam group (treated with diazepam dissolved in soybean oil at a concentration of 0.5 mg/mL), a low-dose SZSO group (SZSO-L, treated with SZSO dissolved in soybean oil at a concentration of 50 mg/mL), and a high-dose SZSO group (SZSO-H, treated with SZSO dissolved in soybean oil at a concentration of 150 mg/mL). The test regents were administered orally at a dosage of 10 mL/kg·bw once daily for a duration of 30 days. After the 30th administration, the locomotor activity frequency of each group was measured using small animal actigraphy for a period of 50 min. Subsequently, a sleep test was conducted following the locomotor activity measurement. Mice in each group received an intraperitoneal injection of 50 mg/kg·bw of sodium pentobarbital (The dosage of sodium pentobarbital was determined through a pre-test, Supplementary Table 1). The onset of sleep was determined by the loss-of-righting reflex, and the duration of their sleep was recorded. For sleep test of SZSO components, forty mice were randomly allocated into four groups: a control group (treated with soybean oil), a SZSO group (SZSO, dissolving SZSO with soybean oil to 100 mg/mL), FA-SZSO group (FA-SZSO, dissolving FA-SZSO with soybean oil to 90 mg/mL), and T-SZSO group (T-SZSO, dissolving T-SZSO with soybean oil to 0.71 mg/mL). The remaining procedures were consistent with above descriptions.

### 2.4 Measurement of neurotransmitters

Following the behavioral tests, the mice were euthanized, and their brains were isolated and placed in centrifuge tubes immersed on ice, followed by the addition of nine times the volume of normal saline solution. After homogenization with a tissue precision homogenizer, the supernatant was obtained through centrifugation (4 °C, 4000 rpm, 10 min). The contents of GABA, 5-HT, NE, Glutamine (Glu), GAD67, GABAAR, SOD, IL-1β, iNOS in the mouse brain homogenate supernatant were examined by assay kit according to the instructions of the manuscripts. The mouse GABA ELISA Kit (Product code: JL12094), GABAAR ELISA Kit (Product code: JL50569), and GAD67 ELISA Kit (Product code: JL46227) were purchased from Shanghai Jianglaibio Science and Technology Ltd., China; The 5-HT assay Kit (Product code: H104), NE assay Kit (Product code: H096), NOS typed assay kit (Product code: A014-1-2), Glu measurement kit (Product code: A074-1-1), IL-1β Assay Kit (Product code: H002), SOD assay kit (Product code: A001-3-2) and Total protein quantitative assay kit (Product code: A045-2-2) were purchased from Nanjing Jiancheng Bioengineering Institute, China.

### 2.5 Saponification of SZSO

100 g of SZSO was placed into a flask with 500 mL of ethanol solution containing potassium hydroxide (1.0 mol/L). The mixture underwent saponification for 5 h before adding 500 mL of deionized water. After cooling, mixture was extracted thrice with petroleum ether (500 mL per extraction). The petroleum ether layer is collected, washed, and separated to obtain T-SZSO. The pH of the lower aqueous phase after petroleum ether extraction was adjusted to 2.0, followed by even stirring and stratification. The upper oily liquid obtained is FA-SZSO.

### 2.6 Component analysis of SZSO

A solution of 50 mg T-SZSO in 5 mL n-hexane was dehydrated using anhydrous sodium sulfate and then filtered through a 0.22 μm filter membrane. Subsequently, a 1 μL sample solution was collected for GC-MS analysis. The chromatographic column used was the InertCap 1 with dimensions of 0.53 mm × 30 m and a particle size of 1.00 μm, provided by GL Sciences Inc. The temperature program started at an initial temperature of 60 °C for 2 min, followed by a rise to 300 °C for 5 min. The inlet temperature was set to 320 °C. Helium was used as the carrier gas with a flow rate of 1.0 mL/min. The splitting ratio was set at 50 to 1. The mass spectrometry parameters consisted of an interface temperature of 280 °C, ion source temperature of 220 °C, MS quadrupole temperature of 140 °C, a solvent delay of 3 min, and a mass scanning range of 35∼540 *m*/*z*. In a 50 mL flask, 0.4 g of FA-SZSO was combined with 6 mL of potassium hydroxide methanol solution and zeolite. The flask containing the mixture was refluxed in a water bath for 10 min, with slow shaking every 30 s. Subsequently, 7 mL of boron trifluoride methanol solution was carefully added to the condensing tube using a pipette. Following the additional reflux for 3 min, 5 mL of iso-octane was added from the top of the condensing tube, followed immediately by the addition of 20 mL of saturated sodium chloride solution. The mixture was vigorously agitated for 15 s and subsequently transferred to a 150 mL separatory funnel for settling. Subsequently, the upper layer solution was decanted into a 25 mL volumetric flask and filled with isooctane up to the mark. An appropriate amount of anhydrous sodium sulfate was added to absorb any trace water in the solution. After agitation, the sample solution was obtained by filtering it through a 0.22 μm membrane. The chromatographic column used was the Aglient J&W Scientific DB-WAX quartz capillary column with dimensions of 30 m × 0.32 mm and a particle size of 0.25 μm. The column temperature was set to 190 °C. The inlet temperature was set to 250 °C. Nitrogen gas (N_2_) was used as the carrier gas with a flow rate of 1.0 mL/min. The detector temperature was set to 250 °C with an air-hydrogen ratio of 10: 1. The splitting ratio was set at 50 to 1.

### 2.7 Network pharmacology analysis

The target genes affected by the active components in T-SZSO were extracted from the Traditional Chinese Medicine Systems Pharmacology Database (TCMSP, https://tcmsp-e.com/tcmsp.php). To ensure the quality of the screening, we set the conditions of oral bioavailability (OB) ≥ 30% and drug-like compound (DL) ≥ 0.1. In order to complement and eliminate redundancy in the obtained target genes, we used the ETCM database (http://www.tcmip.cn/ETCM/index.php/Home/) and SymMap database (www.symmap.org/). Additionally, we searched for sleep-related target genes by using “Sleeping aids” as keywords in the Genecards database (http://www.genecards.org/). Finally, we utilized Venny 2.1 to identify overlapping genes between the T-SZSO target genes and the sleep-related target genes, in order to obtain the direct T-SZSO target genes that promote sleep. The target genes of sleep aids and T-SZSO were listed in Supplementary Table 2.

The intersecting target genes underwent KEGG pathway enrichment analysis, which was performed using the DAVID 2021 database (https://david.ncifcrf.gov/home.jsp). The results were then processed and analyzed using a bioinformatics data processing platform (http://www.bioinformatics.com.cn/en). A visualization network diagram of the Drug-Component-Target-Pathway was constructed using Cytoscape 3.9.0. To further investigate the protein-protein interactions, the intersecting target genes were submitted to the STRING 11.0 database (http://string-db.org/), with *Homo sapiens* selected as the organism. We set a confidence threshold of >0.9 and filtered out unconnected target genes. The resulting TSV-formatted output was optimized using Cytoscape 3.9.0 to generate a protein-protein interaction (PPI) network map and to calculate node information via the MCODE plug-in. This facilitated the identification of hub genes within secondary modules.

### 2.8 Cell culture

Human umbilical vein endothelial cells (HUVECs) were obtained from American Type Culture Collection (ATCC). The cells were cultured with DMEM (containing 10% FBS) (Gibco, Invitrogen, USA) in 5 % CO_2_ incubator (Thermo, USA, 311) at 37 °C.

### 2.9 MTS assay

HUVECs reached about 80% confluence in the logarithmic growth phase were seeded into 96-well plate with a density of 6 × 10^4^ cells per milliliter. 400 μM H_2_O_2_ were added into the culture medium to induce oxidative stress. T-SZSO with different concentrations were added into cells simultaneously and incubated in 5% CO_2_ incubator (Thermo, USA, 311) at 37 °C for 72 h. The viability of HUVECs was determined using CellTiter 96® AQueous One Solution Cell Proliferation Assay (MTS; Promega, Madison, WI, USA). Absorbance was detected using a microplate reader (infiniteM200pro, TECAN, Switzerland).

### 2.10 Statistics

All statistical analyses were performed using IBM SPSS 22. Data were checked for normal distribution (Shapiro–Wilk test) and homogeneity of variance (Levene's test for equality of variance) before choosing the appropriate statistical test. One-way analysis of variance (ANOVA) was used to evaluate the significant difference in normally distributed data. The Kruskal–Wallis test was used for nonnormally distributed data analysis. P values below 0.05 were considered significant for all analyses. All data are presented as the mean ± standard deviation (SD).

## 3 Results

### 3.1 SZSO treatment enhanced the sleep of mice

**Fig. 1:**
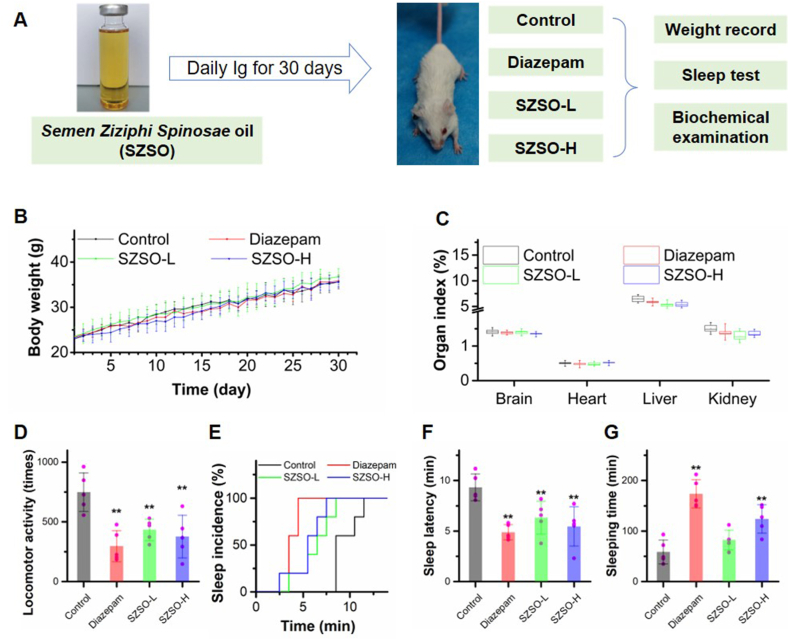
**Effects of SZSO on sleep-promoting. (A)** The representative picture of experimental design. The weight growth curve **(B)**, organ index **(C)**, locomotor activity **(D)**, sleep incidence **(E)**, sleep latency **(F)**, and sleeping time **(G)** of mice after drug administration. **p < 0.01. Error bars indicate SD.

To investigate the effect of SZSO in promoting sleep, we administered high and low doses of SZSO to ICR mice for 30 continuous days. The diazepam treated group was included as a positive control (Fig. 1A). The mice that received SZSO treatment did not exhibit any noticeable changes in terms of weight gain or organ index (Fig. 1B and C). However, the locomotor activities of the SZSO-L and SZSO-H groups were significantly lower than those of the control group, with reductions of 42.1% and 49.7%, respectively (Fig. 1D). Correspondingly, high-dose SZSO significantly increased the incidence of sleep of the mice, reduced the sleep latency, and prolonged their sleep time by 111.1% (Fig. 1E–G). SZSO treated groups showed comparable effects to the diazepam group in reducing locomotor activity and inducing hypnotic effects, indicating its favorable role in promoting sleep.

### 3.2 Identification of T-SZSO as the sleep-promoting components of SZSO

**Fig. 2:**
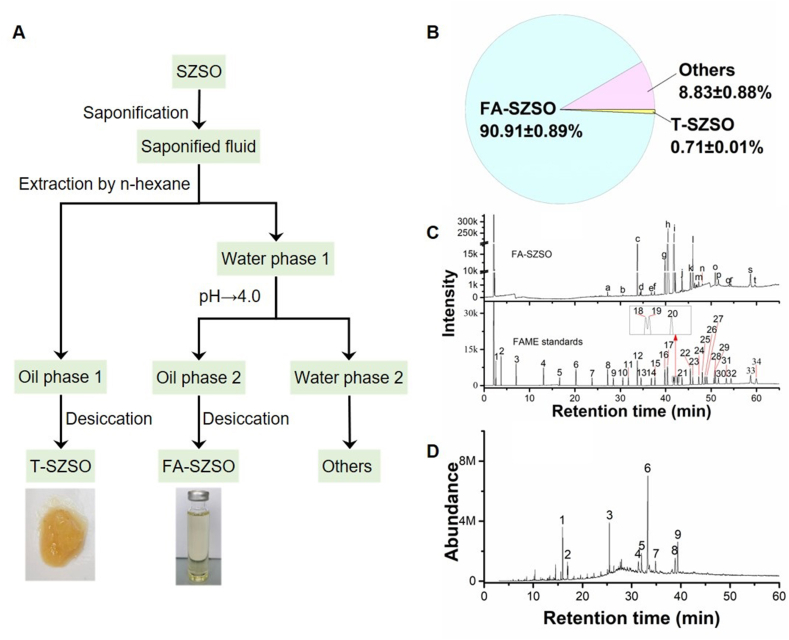
**Separation and component analysis of SZSO. (A)** The flowchart of separation of T-SZSO and FA-SZSO. **(B)** The relative abundance of T-SZSO and FA-SZSO in the total SZSO. **(C)** Gas chromatogram of FA-SZSO and FAME standard. **(D)** Total ion chromatogram of T-SZSO.

In light of the uncertainty of the components of SZSO in promoting sleep, we initially separated FA-SZSO and T-SZSO for further analysis (Fig. 2A). As shown in Fig. 2B, FA-SZSO comprised 90.91% (w/w) and T-SZSO comprised 0.71% (w/w) of total SZSO. Additionally, after undergoing saponification, extraction, and acidification processes, approximately 8.83% (w/w) of the mass of SZSO was found in the residual solution, likely due to the formation of glycerol during triacylglycerol saponification.

The fatty acid composition of SZSO was analyzed using gas chromatography (GC) and the area normalization method (Fig. 2C). Among the 20 main fatty acids identified, oleic acid and linoleic acid collectively accounted for 89.56% (Table 1). Previous studies have demonstrated that the composition of fatty acids in SZSO differs depending on the producing area and extraction method employed, such as pressing, supercritical CO_2_ extraction, and subcritical butane extraction [[20], [21], [22]] [[20], [21], [22]] [[20], [21], [22]]. The saturated fatty acids in SZSO mainly consist of palmitic acid and stearic acid, with a content range of 6%–27%. The unsaturated fatty acids are primarily composed of oleic and linoleic acid, with a content range of 60%–90% [[20], [21], [22], [23], [24], [25]] [[20], [21], [22], [23], [24], [25]] [[20], [21], [22], [23], [24], [25]]. In this investigation, the fatty acid profile and composition of SZSO closely align with the previous results obtained by Guo and Zhou [17,24]. However, it deviates from the outcomes documented by other studies [18,22,23,25].

**Table 1:** Relative contents of fatty acids in FA-SZSO.

Peak labeling		FAME	Relative content (%)
FA-SZSO	Standards		
a	8	Methyl myristate	0.06
b	10	Methyl pentadecanoate	0.02
c	12	Methyl palmitate	3.81
d	13	Methyl palmitoleate	0.10
e	14	Methyl heptadecanoate	0.04
f	15	Methyl *cis*-10-heptadecenoate	0.05
g	16	Methyl stearate	2.06
h	17	Methyl oleate/Methyl elaidate	44.23
i	18	Methyl linoleate/Methyl linolelaidate	45.33
j	21	Methyl arachidate	0.51
k	22	*Cis*-11-eicosenoic acid methyl ester	0.36
l	23	*Cis*-11,14-eicosadienoic acid methyl ester	0.14
m	25	*Cis*-11,14,17-eicosatrienoic acid methyl Ester/*Cis*-8,11,14-eicosatrienoic acid methyl ester	0.02
n	26	Methyl arachidonate	2.65
o	29	*Cis*-13,16-docosadienoic acid methyl ester	0.26
p	30	Methyl tricosanoate	0.10
q	31	Methyl tetracosanoate	0.05
r	32	Methyl *cis*-15-tetracosenoate	0.05
s	33	*Cis*-5,8,11,14,17-eicosapentaenoic acid methyl ester	0.15
t	34	*Cis*-4,7,10,13,16,19-docosahexaenoic acid methyl ester	0.02

Next, gas chromatography-mass spectrometry (GC-MS) was utilized to separate and identify the constituents of T-SZSO. The total ion chromatogram displayed a presence of primarily nine substances in T-SZSO (Fig. 2D). By comparing the mass spectrometric data with the NIST14. L standard spectrum library, the identity of all nine compounds as terpenoids was confirmed (Table 2). These findings align with the analytical results previously reported by Lu [18] and Zhang [19], which identified phytol, geraniol, squalene, campesterol, stigmasterol, γ-sitosterol, and methyl betulinic acid as constituents of SZSO. Notably, the lupeol and betulinic aldehyde were first detected in SZSO in our study.

**Table 2:** Constituent analysis of T-SZSO.

Peak number	R.T. (min)	Terpenoids	Compound	Relative contents (%)	Formula
1	15.958	Chain diterpene	Phytol	12.943	C_20_H_40_O
2	16.956		Geranylgeraniol	6.453	C_20_H_34_O
3	25.502	Chain triterpene	Squalene	16.581	C_30_H_5_0
4	31.355	Tetracyclic triterpenoid	Campesterol	2.290	C_28_H_48_O
5	32.013		Stigmasterol	4.752	C_29_H_48_O
6	33.269		γ-Sitosterol	31.839	C_29_H_50_O
7	34.857	Pentacyclic triterpenoid	Lupeol	3.976	C_30_H_50_O
8	38.831		Methyl betulinic acid	6.724	C_31_H_50_O_3_
9	39.376		Betulinicaldehyde	14.442	C_30_H_48_O_2_

**Fig. 3:**
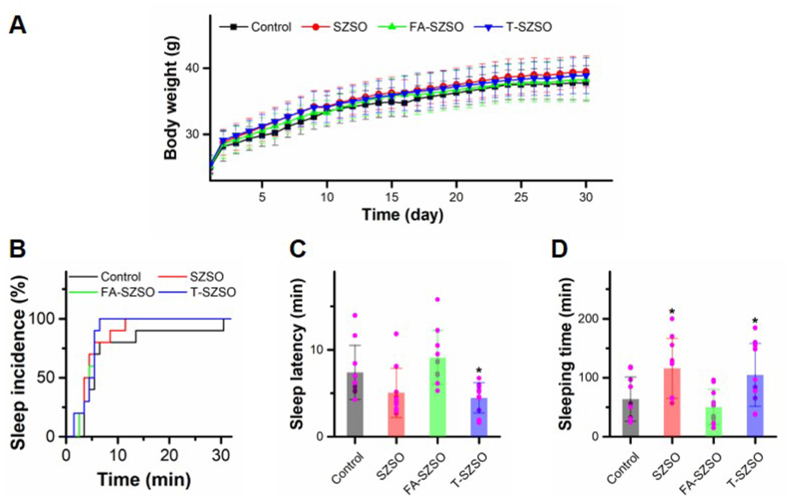
**Active component analysis of SZSO. (A)** The weight growth curve of mice after T-SZSO and FA-SZSO administration. The sleep incidence **(B)**, sleep latency **(C)**, and sleeping time **(D)** of mice after drug administration. *p < 0.05. Error bars indicate SD.

Subsequently, the effects of FA-SZSO and T-SZSO on promoting sleep were investigated through administration of equivalent doses to those present in 100 mg/mL SZSO to mice. Similar to the SZSO treated group, mice treated with FA-SZSO and T-SZSO showed no significant differences in weight gain (Fig. 3A). Strikingly, compared to the control group, treatment with T-SZSO increased sleep incidence, significantly decreased sleep latency, and prolonged sleep time, while FA-SZSO had no effect (Fig. 3B–D). The sleep-promoting effects of T-SZSO is comparable to the SZSO, suggesting that T-SZSO is responsible for the sleep-enhancing effect observed.

### 3.3 Network pharmacology analysis reveals the neuroprotective and neurotransmitter-regulating properties of T-SZSO

**Fig. 4:**
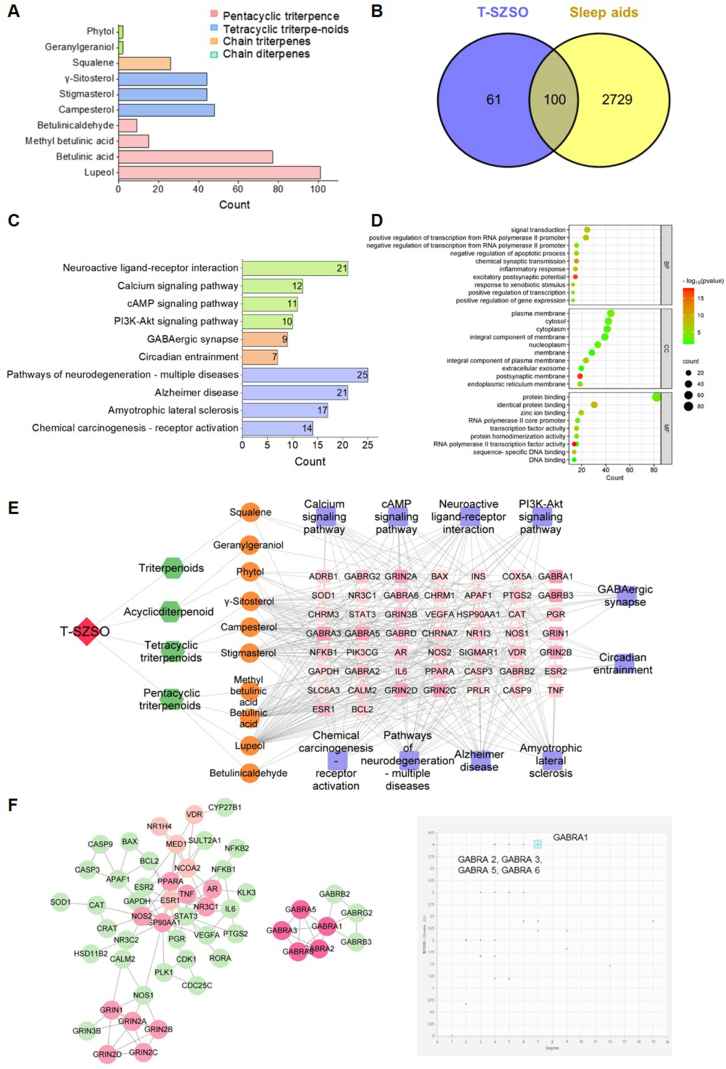
**Network pharmacology analysis of T-SZSO in sleep promoting effect. (A)** The number of targets directly affected by each component in T-SZSO. **(B)** Venn diagram of targets of T-SZSO and sleep aids. **(C)** KEGG pathway enrichment analysis and **(D)** GO analysis. **(E)** Visualization of the “Drug-Component-Target-Pathway” network. Nodes of the same color indicate identical types, and the number of edges indicate the component's importance in the network. **(F)** PPI network. The intensity of color is directly proportional to the MCODE value of the secondary network.

Network pharmacology is a potent method that integrates database bioinformatics and pharmacology to investigate the molecular interactions between drugs and biological systems. By cross-checking the TCMSP database (https://tcmsp-e.com/tcmsp.php), TCMSP (https://tcmsp-e.com/tcmsp.php), and SymMap database (www.symmap.org/), we identified multiple target genes for each terpenoid in T-SZSO, as depicted in Fig. 4A. Compared to chain diterpenoids, chain triterpenoids, and tetracyclic triterpenoids, pentacyclic triterpenoids exhibited a broader range of pharmacological activities, with a total of 202 potential target genes (Fig. 4A). After data deduplication, we obtained a total of 161 direct target genes for T-SZSO. On the other hand, 5357 sleep-promoting target genes were extracted from the Genecards database, and 2829 of them were selected based on the median value of “Gifts”. By intersecting these sets, it was discovered that 62.1% of the targets directly affected by T-SZSO were involved in promoting sleep (Fig. 4B).

The KEGG pathway enrichment analysis revealed that these potentially sleep-aid target genes participate in numerous biological processes such as extracellular and intracellular signaling transduction, GABAergic synapse, and circadian entrainment (Fig. 4C). Interestingly, some of the target genes are also potentially involved in the pathogenesis of neuronal diseases. For example, there are 21 genes related to Alzheimer's disease, which is consistent with the theory that sleep disorders contribute profoundly to the neurodegeneration process (Fig. 4C). GO functional enrichment analysis was performed on a set of 100 intersecting genes (Fig. 4D). The target genes associated with T-SZSO exhibited significant enrichment in 298 biological processes, encompassing signal transduction, chemical synaptic transmission, post-synaptic membrane potential, response to external stimuli, and several others. Moreover, these genes showed enrichment in 62 cellular components, such as GABA-A receptor complexes, presynaptic membranes, dendritic spines, myelin sheaths, neuronal projections, among others. Furthermore, 87 molecular functions were found to be enriched, including protein binding, involvement of neurotransmitter-gated ion channel activity in the regulation of post-synaptic membrane potential, neurotransmitter receptor activity, GABA-A receptor activity, and excitatory extracellular ligand-gated ion channel activity (Fig. 4D).

Fig. 4E illustrated the network relationships among T-SZSO, active components, target genes, and related pathways. Several critical genes in the oxidative pathway (SOD1, CAT) and inflammation pathway (NFκB, TNFα, IL-6) were identified. Moreover, a group of GRIN genes that are involved in the regulation of circadian and synapses plasticity were also listed, suggesting a potential role of T-SZSO in enhancing memory and learning ability. Notably, the two newly identified pentacyclic triterpenoids, lupeol and betulinicaldehyde, had the most interaction with the target genes, indicating an important role of these two pentacyclic triterpenoids in the pharmacological effectiveness of T-SZSO. To better understand the regulation relationship between these target genes, we conducted protein-protein interaction (PPI) network analysis (Fig. 4F). The result suggested that there were no genes other than GABRs involved in the regulation of GABAergic synapse, and GABRAs were hub genes in this network. MCODE analysis demonstrated the central role of GABRA1, GABRA2, GABRA3, GABRA5, and GABRA6 genes in T-SZSO's sleep-enhancing effects. Among them, GABRA1 had a degree of 7 and a MCODE score of 4.

### 3.4 T-SZSO modulates neurotransmitter signaling and alleviates cellular stress

**Fig. 5:**
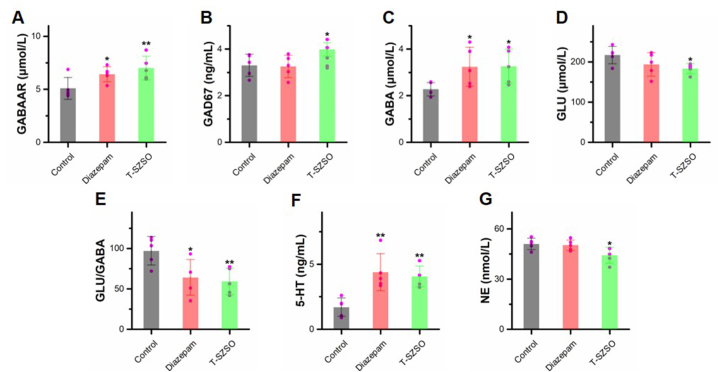
**T-SZSO regulates neurotransmitter signaling in mice. (A**–**G)** The protein levels of GABAAR, GAD67, GABA, GLU, 5-HT, and NE in T-SZSO treated mice brain. *p < 0.05, **p < 0.01. Error bars indicate SD.

To confirm the speculated mechanisms of T-SZSO in promoting sleep, we examined the levels of proteins involved in inhibitory and excitatory neurotransmitter signaling in the mouse brain. The GABARA, as shown in the network pharmacology analysis, was upregulated in the T-SZSO treated group (Fig. 5A). Furthermore, GABA and its major synthetic enzyme GAD67, were also up-regulated following T-SZSO treatment (Fig. 5B and C). Interestingly, the positive drug increased the content of GABA without affecting the GAD67 level, which probably inhibited the GABA degradation pathway. Conversely, the level of excitatory neurotransmitter glutamine and the ratio of glutamine/GABA was reduced (Fig. 5D and E). Additionally, although not identified by the network pharmacology analysis, the level of 5-HT was significantly elevated, and the NE was down-regulated in the T-SZSO treated mice brain (Fig. 5F and G). Previous studies suggested that SZSO may also participate in the regulation of serotonin secretion [38]. Our finding further supported the previous report.

**Fig. 6:**
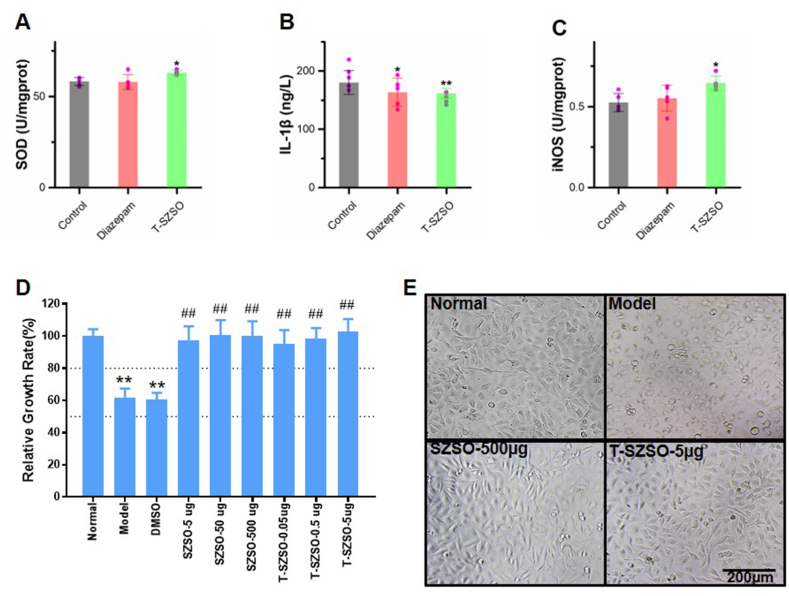
**T-SZSO alleviates cellular stress. (A**–**C)** The protein levels of SOD, IL-1β, and iNOS in T-SZSO treated mice brain. **(D, E)** MTS assay of HUVEC cell under oxidative stress. *, #p < 0.05, **, ##p < 0.01. Error bars indicate SD.

In addition to regulating neurotransmitters, T-SZSO also potentially exerts anti-oxidative stress and anti-inflammation effects, as suggested by network pharmacology analysis. We examined the levels of SOD, IL-1β, and iNOS in the brains of mice treated with T-SZSO. The ELISA results indicated that T-SZSO upregulated the levels of SOD and iNOS, while decreasing IL-1β (Fig. 6A–C). Interestingly, the positive control diazepam only decreased the level of IL-1β and had no effect on SOD and iNOS.

To further confirm the effects of T-SZSO on oxidative stress and inflammation, we performed an MTS assay to assess the cell viability of HUVEC cells under oxidative stress. The results showed that both SZSO and T-SZSO rescued cell viability in a dose-dependent manner (Fig. 6D and E). In summary, these findings demonstrate the important roles of T-SZSO in promoting sleep and anti-oxidative stress.

## 4 Discussion

Sleep aids derived from natural plants have emerged as popular choices for enhancing sleep quality due to their exceptional safety and efficacy. Numerous individual herbs, such as SZS, *Acanthopanax senticosus*, *Ganoderma lucidum*, and *Poria cocos*, exhibit positive effects on sleep improvement. Moreover, several classical Chinese medicine prescriptions like AnShenBuNaoYe and Suanzaoren decoction have demonstrated significant impacts on sleep enhancement. However, the active ingredients responsible for improving sleep in these natural substances remain unclear, along with their interactions and changes during processing. Furthermore, understanding how these substances exert their effects after entering the body is essential to further advance the application of traditional Chinese medicine in promoting better sleep. *Semen Ziziphi Spinosae* oil (SZSO) is a mixture of lipid components, mainly composed of triglycerides and liposoluble secondary metabolites, accounting for over 30% of SZS [[17], [18], [19]] [[17], [18], [19]] [[17], [18], [19]]. This study represented the first exploration of the sleep-promoting mechanism and active components of SZSO. It revealed that liposoluble secondary metabolites, mainly terpenoids, enhanced GABA signaling and regulate neurotransmitters in the brain, and had an oxidative stress suppression effect. These findings had implications for enhancing the quality of related products and promoting the acceptance of natural sleep-promoting agents.

Previous studies have shown the importance of trace liposoluble secondary metabolites found in plant oils, such as vitamin E, polyphenols, squalene, plant sterols, hydrocarbons, and glycoside ligands, in performing specific functional activities [39]. Terpenoids have widely documented therapeutic effects. For example, citral and pinene, which are the main active ingredients in many aromatic plant oils, are known for their sedative, anxiolytic, and central nervous system inhibitory effects [40,41]. Terpenoids extracted from *T. erecta* [42], *Lippia graveolens Kunth* [43], and leaves of *Carissa carcarandas* [44] are also the main substances responsible for their anti-anxiety, sedative, and hypnotic activities. This study provided a scientific explanation and basis for the sleep-promoting effect of this class of substances.

SZSO comes from SZS, thus establishing a connection between the two in terms of their sleep-promoting mechanisms. Existing research has shown that, the composition of SZS is diverse, and its mechanism for treating insomnia involves the integration of multiple components, targets, and pathways [45]. Saponins, flavonoids, alkaloids, and other components have been identified as the main sedative components of SZS [[46], [47], [48], [49]] [[46], [47], [48], [49]] [[46], [47], [48], [49]]. The actions of each component are not completely identical. For example, SZS saponins increase the level of GABA in the brain, reduce the content of NE in the brain, and induce an increase in the expression level of GABAAR genes, thereby affecting sleep [[50], [51], [52], [53]] [[50], [51], [52], [53]] [[50], [51], [52], [53]]. Spinosin, the main flavonoid component in SZS, can prevent neuronal apoptosis caused by acrylamide and its activity in improving sleep is closely related to the 5-HT system [49,54]. The alkaloid in SZS enhances the expression of glutamate decarboxylase and activates GAD65/67, indirectly influencing sleep by increasing GABA content. It also activates GABAAR and promotes the binding of GABA to the receptor [[55], [56], [57]] [[55], [56], [57]] [[55], [56], [57]]. The findings of our research indicate that the mechanisms of sleep promotion by T-SZSO and SZS saponins are very similar. Both mechanisms are related to the regulation of GABA signaling pathway and neurotransmitter levels. T-SZSO promotes sleep by significantly increasing the levels of GABA, GABAAR, and 5-HT in the mouse brain, and reducing the levels of NE and GLU.

The biological activity is exhibited in accordance with the compound's structural characteristics. The sleep-promoting mechanism of SZSO and SZS saponins exhibited similarity, suggesting their resemblance in terms of substance composition and structure. The direct penetration of saponin A into the brain has been demonstrated to be limited [54], with a measured bioavailability of only 1.32% in rats [58]. The aglycones, as a metabolites of SZS saponins, exert a significant influence on the expression and activation of GABAAR, rather than the SZS saponins [50]. Compared to SZS saponin, the aglycones has a better affinity with GABAAR [59,60], better blood-brain barrier permeability, and is more easily absorbed by the human body [61,62]. In this study, network pharmacological analysis was employed to investigate the targets and pathways of the components in T-SZSO. The findings revealed that GABRAs served as the central gene in the target network of T-SZSO, with GABRA1, GABRA2, GABRA3, GABRA5, and GABRA6 genes playing a pivotal role in enhancing sleep. GABA is a crucial inhibitory neurotransmitter in the brain that interacts with three types of receptors: A, B, and C [63]. Among these receptors, extensive attention has been given to GABAA type receptors in insomnia research [9]. Upon binding with its receptor, GABA enhances chloride permeability across nerve cell membranes [64]. This influx of chloride ions induces membrane hyperpolarization [65] and subsequently reduces excitability levels while producing sedative, hypnotic and anxiolytic effects [66]. Amongst the various subunits of the GABAA receptor family implicated in insomnia mediation are frequently reported ones such as GABRA1 and GABRG2 [67,68]. These results aligned consistently with those obtained from PPI network calculations as well as MCODE nodes analysis. Furthermore, upregulation of mouse brain's expression of gamma-aminobutyric acid type A (GABAAR) receptors by SZSO further validated these findings. Therefore, targeting pathways associated with GABA holds great promise for advancing our understanding on how SZSO promotes sleep. Further, the network pharmacology studies have shown a greater correlation between pentacyclic triterpenoids in T-SZSO and sleep-enhancing target proteins and pathways, followed by tetracyclic triterpenoids. The active ingredients of T-SZSO, pentacyclic triterpenoids, and aglycones of SZS saponins have similar structures [69,70]. In addition, sterol glycosides in nuts or seeds can release free plant sterols when hydrolyzed, which facilitates absorption by plant oil containing triglycerides [71]. Therefore, it is reasonable to assume that the pentacyclic triterpenoids found in T-SZSO may originate from the degradation and transformation of SZS saponins. After saponins are transformed into monomers, their lipophilicity is enhanced, and they dissolve into T-SZSO. These studies indicate that T-SZSO has an advantage in exerting sleep-promoting activity due to its higher content of non-saponifiable lipophilic components.

Furthermore, research studies have demonstrated that sleep deprivation can induce oxidative stress in rats or mice [72,73], and neuronal cells are easily damaged by oxidative stress [74]. The circulatory system is also vulnerable to the detrimental effects of oxidative stress. Patients with insomnia exhibit impaired vascular endothelial function [75]. Particularly during sleep deprivation, endothelial cells can activate microglia and further result in neuronal damage through oxidative stress mechanisms [76]. HUVECs cells were employed to investigate whether SZSO or T-SZSO could alleviate oxidative stress and validate their role in protecting endothelial cells. The findings revealed a significantly higher relative growth rate of cells in the SZSO and T-SZSO groups compared to the oxidative stress model group. These results provided evidence supporting the neuroprotective effect of SZSO or T-SZSO by ameliorating cellular oxidative stress.

The present study has yielded valuable findings regarding the active components and mechanism of SZSO in promoting sleep; however, certain limitations still exist.

First of all, numerous Chinese herbal medicines have demonstrated effectiveness in improving sleep, including SZS, *Acanthopanax senticosus*, *Poria cocos*, *Schisandrae fructus*, and *Eucommia ulmoides*. Previous studies have shown that SZS (2.7 g/kg·bw, administered continuously for 7 days) significantly increase the sleep duration of PCPA-induced insomnia rats by nearly 100% and reduce the sleep latency by 66% [9]. *Acanthopanax senticosus* (16 g/kg·bw, administered continuously for 7 days) extend the sleep time of mice by 210% [77]. The water extract of *Zhuporia cocos* (4.5 g/kg·bw, administered continuously for 7 days) prolong the sleep duration by 230% [78]. Additionally, both water and ethanol extracts of *Schisandra chinensis* (2.7 g/kg·bw each; single administration) increase the sleep time in mice by approximately 160% and 200%, respectively [79]. Furthermore, *Eucommia* water extract (0.6 g/kg·bw; continuous administration for 30 days) enhance sleep duration by 157% while reducing sleep latency by15% [11]. Combinations of different Chinese herbs have also exhibited notable effects in promoting better quality sleep. For instance, SZS combined with *Schisandra chinensis* synergistically treats insomnia [10]. The blend of SZS and GABA demonstrated an amelioration in sleep quality among mice while effectively addressing metabolic imbalances associated with sleep disorders [80]. Our study demonstrated that the administration of SZSO at a dosage of 0.15 g/kg·bw for a duration of 30 days significantly increased sleep duration by 211%, reduced sleep latency by 41%, and decreased voluntary activity levels by 50%. The data comparison revealed that SZSO exhibited a remarkable efficacy in promoting sleep. However, further experimental verification is required to compare its efficacy with other medicinal substances.

Secondly, this study validated the sleep-promoting activity and mechanism of SZSO and its terpenoids in mice. However, further clinical trials are required to confirm their efficacy in promoting sleep in humans. Previous studies have demonstrated the significant clinical benefits of SZS in treating insomnia. Daily consumption of 15 g SZS has been shown to alleviate clinical symptoms, such as anxiety and depression in elderly patients with insomnia thus improving their sleep quality [81]. Another study also indicated that 15 g/day SZS free decoction granules significantly enhanced sleep quality and state among patients [82]. Therefore, it is reasonable to assume that SZSO may possess a similar sleep-promoting effect in humans. Nevertheless, this assumption needs validation through clinical trials. Key issues and potential challenges that should be considered during this confirmation process include: (1) Potential differences in the biological mechanisms of sleep between humans and mice, imply that strategies effective for mice may not necessarily work for humans. (2) Drug metabolism and dosage considerations: The rate of drug metabolism differs between mice and humans, necessitating precise adjustments to ensure equivalent concentrations as used in mouse experiments. (3) Long-term effects: Animal studies can be observed effects over relatively short periods, while human subjects may require longer durations for similar effects to manifest.

Once again, network pharmacological analysis was employed to investigate the targets and pathways of the components in T-SZSO, aiming to compare their correlation with previous experimental studies on neurotransmitters and the GABA signaling pathway. This analysis provided preliminary guidance for subsequent screening, isolation, identification, and mechanism research of effective components in SZSO to enhance sleep quality. However, it should be noted that network pharmacological analysis has certain limitations. On one hand, the field of network pharmacology relies on databases and analytical methods, but its reliability is contingent upon the accuracy of data and the standardization of analytical techniques, necessitating further experimental validation. On the other hand, it fails to reflect the dose-response relationship between pharmacodynamic substances and efficacy since drug dosage plays a crucial role in achieving therapeutic effects. Therefore, animal or cell experiments are required to validate the separation of each component within T-SZSO as well as establish its dose-response relationship.

Finally, this study has yielded valuable findings in the investigation of the active constituents and mechanism of SZSO in promoting sleep. However, certain limitations still exist. While the somnogenic activity of SZSO has been validated in mice, further clinical trials are imperative to substantiate its hypnagogic effect on humans. Moreover, although T-SZSO serves as the pivotal sleep-enhancing component within SZSO, it remains a blend of multiple fat-soluble non-saponifiable constituents. Therefore, subsequent studies should focus on more intricate separation techniques for T-SZSO and conduct comprehensive evaluations regarding the sleep-promoting or neuroprotective functions of each individual constituent. Due to the low content of several components in T-SZSO and the difficulty in separation, further work is needed to improve the separation method and activity verification.

## 5 Conclusions

*Semen Ziziphi Spinosae* oil (SZSO) was a natural vegetable oil with potent sleep-promoting effects, and the terpenoids present in SZSO (T-SZSO) played a pivotal role in facilitating its soporific properties. Our findings provided the initial evidence of T-SZSO's sleep-enhancing effects through a mechanism involving neurotransmitter regulation and anti-oxidative stress. The pentacyclic triterpenoids present in SZSO might serve as the key to enhance sleep quality.

## Data availability statement

Data included in article/supp. material/referenced in article.

## Ethics statement

The animal protocols were approved by the Ethics Committee of Hebei Yiling Pharmaceutical Research Institute (approval number: N2022089), and all procedures were executed according to the Technical Standards for the Testing & Assessment of Health Food (2003).

The ethical review of animal testing is as follows:

## CRediT authorship contribution statement

**Mingzhe Sun:** Writing – review & editing, Writing – original draft, Validation, Project administration, Methodology, Investigation, Formal analysis, Data curation, Conceptualization. **Mengnan Li:** Writing – review & editing, Validation, Methodology, Investigation, Data curation, Conceptualization. **Xinwen Cui:** Validation, Methodology, Investigation, Formal analysis. **Lin Yan:** Validation, Investigation, Formal analysis. **Yiqiao Pei:** Validation, Investigation, Formal analysis. **Chao Wang:** Validation, Methodology, Formal analysis. **Chunbo Guan:** Validation, Investigation, Formal analysis. **Xiuqing Zhang:** Writing – review & editing, Supervision, Resources, Formal analysis, Conceptualization.

## Declaration of competing interest

The authors declare that they have no known competing financial interests or personal relationships that could have appeared to influence the work reported in this paper.

## Abbreviations

SZS: Semen Ziziphi Spinosae
SZSO: Semen Ziziphi Spinosae oil
T-SZSO: Terpenoids of SZSO
FA-SZSO: Fatty acids of SZSO
SZSO-L: Low-dose SZSO group
SZSO-H: High-dose SZSO group.
