# Supplementary material for: Terpenoids derived from Semen Ziziphi Spinosae oil enhance sleep by modulating neurotransmitter signaling in mice
Source: Heliyon. 2024 Mar 1;10(5):e26979. doi: 10.1016/j.heliyon.2024.e26979 (PMC10923681; doi:10.1016/j.heliyon.2024.e26979)
Supplement: Multimedia component 1 [file mmc1.doc]

# Supplementary material

## 1 The pentobarbital sodium dosage screening test

The advanced pre-test preceding the formal experiment was conducted to ensure that all animals achieved 100% sleep induction, while maintaining an optimal sleep duration. Pentobarbital sodium (30-60 mg/kg·BW) was administered for the formal experiment. Pre-test results (Table 1) demonstrated that doses of pentobarbital sodium at 40, 50, and 60 mg/kg·bw induced complete sleep-in mice. However, the dose of 40 mg/kg·bw resulted in a too short sleep duration and potential instances of failure to induce sleep, whereas a dose of 60 mg/kg·bw led to excessively prolonged sleep, compromising test efficiency. Therefore, this study selected a moderate dosage of pentobarbital sodium (50 mg/kg·bw), allowing mice to achieve a suitable duration of approximately 40 minutes for the formal testing.

Table 1 Pre-test results of sodium pentobarbital dose screening

| Mouse No. | Pentobarbital sodium dosage（mg/kg·bw） | Sleep incidence（%） | Sleep latency（min） | Average sleep latency（min） | Sleeping time（min） | Average sleep time（min） |
| --- | --- | --- | --- | --- | --- | --- |
| 1 | 40 | 100 | 5.10 | 6.70 | 24.28 | 15.17 |
| 2 |  |  | 8.40 |  | 3.63 |  |
| 3 |  |  | 6.60 |  | 17.60 |  |
| 4 | 50 | 100 | 6.75 | 5.07 | 41.13 | 42.06 |
| 5 |  |  | 4.87 |  | 43.87 |  |
| 6 |  |  | 3.58 |  | 41.17 |  |
| 7 | 60 | 100 | 3.00 | 2.92 | 83.47 | 85.82 |
| 8 |  |  | 2.67 |  | 97.40 |  |
| 9 |  |  | 3.08 |  | 76.60 |  |

## 2 The target genes and of sleep aid and T-SZSO

Table 2 Gene list

| T-SZSO intersects with disease genes | T-SZSO does not interact with disease genes | Sleep aids |
| --- | --- | --- |
| **100** | **61** | **2829** |
| AR | ACAT2 | FGFR1 |
| PTGS2 | ADC | FGFR3 |
| ACAT1 | ADH1A | FGFR2 |
| CAT | ADH1C | MTOR |
| CETP | ADRA2C | EGFR |
| COASY | AKR1C1 | RET |
| DHCR7 | AKR1C2 | MMP9 |
| FDPS | CATSPER1 | NTRK2 |
| GAPDH | CCNB1 | AKT1 |
| INS | CDC2 | TP53 |
| LDLR | CDK2 | STAT3 |
| MVD | CES1 | ESR1 |
| MVK | CLEC4E | TGFBR1 |
| SORT1 | COX4I1 | MAP2K1 |
| SQLE | COX5B | IGF1R |
| TLR1 | COX6A2 | ERBB4 |
| TLR3 | COX6B1 | AKT3 |
| NCOA2 | COX6C | MAP2K2 |
| MAOB | COX7A1 | CDK4 |
| NR1H4 | COX7B | RAF1 |
| ACHE | COX7C | MET |
| ALB | COX8A | HDAC6 |
| CHRM1 | DHRS1 | SOD1 |
| CHRM3 | DHRS2 | PSEN1 |
| ESR1 | DHRS3 | SLC2A1 |
| GABRA1 | DHRS7 | CTNNB1 |
| NOS2 | EFTUD1 | MAPK1 |
| PPARA | FABP6 | PPARG |
| TNF | FDFT1 | CREBBP |
| DPP4 | FGFR4 | MMP2 |
| CYP27B1 | GABRA4 | ATM |
| MED1 | GABRB1 | TGFB1 |
| NR1I3 | GABRE | BRAF |
| RORA | GABRG1 | NFKB1 |
| VDR | GABRG3 | MPO |
| ESR2 | GABRP | PTPN11 |
| GABRA2 | GABRQ | STAT1 |
| GABRA3 | GPBAR1 | KDR |
| PGR | GRIN3A | PDGFRA |
| GABRA5 | HOXA10 | INSR |
| GABRA6 | HSD17B1 | PDGFRB |
| GABRB2 | IGHG2 | BTK |
| GABRB3 | IVD | CDK5 |
| GABRD | LIP3 | ERBB3 |
| GABRG2 | LSS | IDH1 |
| GRIN1 | MIA | JAK1 |
| GRIN2A | MT-CO1 | SMAD3 |
| GRIN2B | MT-CO2 | PIK3CD |
| GRIN2C | MT-CO3 | ADAM10 |
| GRIN2D | NCOA1 | TYK2 |
| GRIN3B | NOX1 | ATR |
| NR1I2 | OXR1 | EPHA2 |
| NR3C1 | PMVK | GRIN2A |
| NR3C2 | PPO2 | IL6 |
| SIGMAR1 | PRSS1 | TNF |
| SULT2A1 | PTPN1 | SNCA |
| PTGS1 | RXRA | ACE |
| HSP90AA1 | SFN | COMT |
| PIK3CG | SGK1 | DNMT1 |
| NPPB | SULT2B1 | CXCR4 |
| ADRB1 | TIPARP | MYC |
| SLC6A2 |  | UCHL1 |
| SLC6A3 |  | GRIN2B |
| HSD11B1 |  | CASP8 |
| COX5A |  | DBH |
| ESRRG |  | HRAS |
| FECH |  | EP300 |
| GSTP1 |  | CAT |
| HSD11B2 |  | HDAC4 |
| NFKB1 |  | AR |
| NFKB2 |  | TLR2 |
| PLA2G1B |  | TERT |
| PRLR |  | LDLR |
| ADH1B |  | PIK3CA |
| LYZ |  | CTSD |
| MAOA |  | HMOX1 |
| CALM2 |  | PTEN |
| CHRNA7 |  | CA2 |
| BCL2 |  | RPS6KA3 |
| BAX |  | CAMK2A |
| CASP9 |  | PRKACA |
| CASP3 |  | RELA |
| SOD1 |  | PTPRC |
| CDK1 |  | HSPB1 |
| CDC25C |  | LCK |
| APAF1 |  | NT5E |
| CA2 |  | FLT4 |
| CRAT |  | TGFBR2 |
| IL6 |  | PLAU |
| KLK3 |  | KRAS |
| KRT16 |  | ITGB3 |
| NOS1 |  | NOTCH1 |
| ODC1 |  | CDH2 |
| PLK1 |  | PRKCD |
| POLD1 |  | NFKB2 |
| STAT3 |  | CTSB |
| TYR |  | MDM2 |
| UNG |  | ABL1 |
| VEGFA |  | HDAC2 |
| DPEP1 |  | SMAD2 |
|  |  | BCR |
|  |  | PPP3CA |
|  |  | CSF1R |
|  |  | PLCG2 |
|  |  | DPYD |
|  |  | SMAD4 |
|  |  | CBL |
|  |  | NOTCH2 |
|  |  | NTRK3 |
|  |  | ACVR1 |
|  |  | TLR3 |
|  |  | TH |
|  |  | GRIA3 |
|  |  | NOTCH3 |
|  |  | APP |
|  |  | TFRC |
|  |  | PARP1 |
|  |  | PDGFB |
|  |  | TLR4 |
|  |  | HSP90AA1 |
|  |  | NGF |
|  |  | SMARCA4 |
|  |  | EGF |
|  |  | MAPK14 |
|  |  | TGFB2 |
|  |  | IFNGR1 |
|  |  | NFE2L2 |
|  |  | SRC |
|  |  | VIM |
|  |  | CFTR |
|  |  | ELANE |
|  |  | JAG1 |
|  |  | AGTR1 |
|  |  | CD19 |
|  |  | ALK |
|  |  | PRKCG |
|  |  | ZAP70 |
|  |  | HGF |
|  |  | CHUK |
|  |  | HSD11B1 |
|  |  | CALR |
|  |  | DNMT3A |
|  |  | TEK |
|  |  | MYLK |
|  |  | GRIA2 |
|  |  | ALPL |
|  |  | CASR |
|  |  | PROC |
|  |  | PLK1 |
|  |  | ATP2A2 |
|  |  | NPM1 |
|  |  | ADAM17 |
|  |  | MAP3K7 |
|  |  | AURKB |
|  |  | APOE |
|  |  | IFNG |
|  |  | KAT5 |
|  |  | MAPT |
|  |  | LRRK2 |
|  |  | ICAM1 |
|  |  | SLC6A3 |
|  |  | CD4 |
|  |  | FOS |
|  |  | B2M |
|  |  | GRIA1 |
|  |  | KCNQ1 |
|  |  | GRIN1 |
|  |  | ADA |
|  |  | DDC |
|  |  | SCN5A |
|  |  | VCP |
|  |  | SHH |
|  |  | LEPR |
|  |  | SERPINE1 |
|  |  | ABCB1 |
|  |  | CD40LG |
|  |  | IL2RA |
|  |  | KCNK3 |
|  |  | APOA1 |
|  |  | CTSK |
|  |  | TNFRSF1A |
|  |  | IL6R |
|  |  | PTCH1 |
|  |  | IL2RB |
|  |  | VDR |
|  |  | KCNH2 |
|  |  | TF |
|  |  | BCL2 |
|  |  | BCHE |
|  |  | BRCA1 |
|  |  | FLT1 |
|  |  | CBS |
|  |  | GSK3B |
|  |  | FAS |
|  |  | NFKBIA |
|  |  | BAX |
|  |  | CD40 |
|  |  | IL1RN |
|  |  | FTH1 |
|  |  | MME |
|  |  | DPP4 |
|  |  | CXCR2 |
|  |  | MAPK8 |
|  |  | GAPDH |
|  |  | DYRK1A |
|  |  | CD36 |
|  |  | PCSK9 |
|  |  | GRM1 |
|  |  | ITGB2 |
|  |  | PRKDC |
|  |  | ALDH2 |
|  |  | FN1 |
|  |  | G6PD |
|  |  | GJA1 |
|  |  | DNM1 |
|  |  | CACNA1H |
|  |  | TYMS |
|  |  | RHOA |
|  |  | NTRK1 |
|  |  | GLUD1 |
|  |  | GSTP1 |
|  |  | PRKCA |
|  |  | HDAC1 |
|  |  | ITGB1 |
|  |  | GNAS |
|  |  | PRKG1 |
|  |  | DNMT3B |
|  |  | RARA |
|  |  | STAT6 |
|  |  | AHCY |
|  |  | MMP1 |
|  |  | FZD4 |
|  |  | RPS6KA1 |
|  |  | ADK |
|  |  | STAT5B |
|  |  | TUBB |
|  |  | PCNA |
|  |  | UGT1A1 |
|  |  | SMARCA2 |
|  |  | LDHA |
|  |  | DSP |
|  |  | EPAS1 |
|  |  | SERPINC1 |
|  |  | CSNK2A1 |
|  |  | MMP3 |
|  |  | SCD |
|  |  | CDC42 |
|  |  | TBXA2R |
|  |  | RAD51 |
|  |  | ATP1A1 |
|  |  | FOXO1 |
|  |  | CACNA1G |
|  |  | TACR3 |
|  |  | CAPN1 |
|  |  | LRP5 |
|  |  | F10 |
|  |  | WNT5A |
|  |  | TNFRSF10B |
|  |  | SOS1 |
|  |  | NRAS |
|  |  | PTH1R |
|  |  | KRT18 |
|  |  | MMP14 |
|  |  | MMP13 |
|  |  | PHGDH |
|  |  | LAMB1 |
|  |  | BMPR1A |
|  |  | SLC9A1 |
|  |  | ENPP1 |
|  |  | CCL2 |
|  |  | TUBA1A |
|  |  | GBA1 |
|  |  | BDNF |
|  |  | CHRNA2 |
|  |  | ALB |
|  |  | VEGFA |
|  |  | NOS3 |
|  |  | CHRNA4 |
|  |  | DRD2 |
|  |  | AGT |
|  |  | MAOA |
|  |  | CACNA1C |
|  |  | SNAP25 |
|  |  | SPR |
|  |  | NR3C1 |
|  |  | GABRB3 |
|  |  | NOS2 |
|  |  | GDNF |
|  |  | SCN2A |
|  |  | TPH2 |
|  |  | SCN8A |
|  |  | RORA |
|  |  | CYP3A4 |
|  |  | GLI2 |
|  |  | USP7 |
|  |  | GAA |
|  |  | STXBP1 |
|  |  | FKBP5 |
|  |  | CACNA1A |
|  |  | EDNRA |
|  |  | MAPK3 |
|  |  | NPC1 |
|  |  | PSEN2 |
|  |  | LMNA |
|  |  | TNFRSF1B |
|  |  | IGF2 |
|  |  | CASK |
|  |  | TBK1 |
|  |  | SLC1A3 |
|  |  | TSC2 |
|  |  | NOS1 |
|  |  | SYK |
|  |  | SMPD1 |
|  |  | MAPK10 |
|  |  | CASP3 |
|  |  | RIPK1 |
|  |  | TRPV4 |
|  |  | SQSTM1 |
|  |  | RAC1 |
|  |  | CREB1 |
|  |  | GSR |
|  |  | PGR |
|  |  | GAD1 |
|  |  | PTGS2 |
|  |  | EDNRB |
|  |  | IL6ST |
|  |  | GGT1 |
|  |  | SIRT1 |
|  |  | GSN |
|  |  | CYBB |
|  |  | LIMK1 |
|  |  | EIF2AK2 |
|  |  | KCNMA1 |
|  |  | LPL |
|  |  | GNAQ |
|  |  | SLC1A2 |
|  |  | AIFM1 |
|  |  | APC |
|  |  | CASP1 |
|  |  | SERPINA1 |
|  |  | F2 |
|  |  | DNM2 |
|  |  | GLA |
|  |  | GRIN2D |
|  |  | GRIK2 |
|  |  | ACAT1 |
|  |  | HK1 |
|  |  | TNFSF11 |
|  |  | ASAH1 |
|  |  | GABRB2 |
|  |  | EIF4E |
|  |  | PLA2G7 |
|  |  | NRG1 |
|  |  | HSPA8 |
|  |  | COL1A1 |
|  |  | ACE2 |
|  |  | GCK |
|  |  | C3 |
|  |  | FBP1 |
|  |  | YWHAE |
|  |  | PCSK1 |
|  |  | MSH2 |
|  |  | GLS |
|  |  | SLC12A2 |
|  |  | GRIA4 |
|  |  | NR5A1 |
|  |  | NPR2 |
|  |  | SLC25A4 |
|  |  | PLAT |
|  |  | CDKN1A |
|  |  | COL2A1 |
|  |  | HDAC9 |
|  |  | GATA2 |
|  |  | FCGR2B |
|  |  | PIK3C3 |
|  |  | GATA3 |
|  |  | MST1R |
|  |  | IRAK1 |
|  |  | CACNA1B |
|  |  | PLG |
|  |  | RAB7A |
|  |  | CPS1 |
|  |  | ACTB |
|  |  | MYB |
|  |  | EPCAM |
|  |  | ESR2 |
|  |  | CD55 |
|  |  | PRKD1 |
|  |  | UBA1 |
|  |  | PRKCQ |
|  |  | MYD88 |
|  |  | PDE3A |
|  |  | CPT2 |
|  |  | DICER1 |
|  |  | DAPK1 |
|  |  | SLC2A2 |
|  |  | ALDH1A2 |
|  |  | RPS6KB1 |
|  |  | SLC6A9 |
|  |  | PRKCH |
|  |  | SPARC |
|  |  | PRMT1 |
|  |  | P4HB |
|  |  | GLI1 |
|  |  | VLDLR |
|  |  | POR |
|  |  | ZEB1 |
|  |  | ACTN1 |
|  |  | IFNAR2 |
|  |  | PRKAG2 |
|  |  | RUNX1 |
|  |  | POLE |
|  |  | PPP2CA |
|  |  | HNF4A |
|  |  | STK4 |
|  |  | KAT2A |
|  |  | RAD50 |
|  |  | ARG1 |
|  |  | ITGB4 |
|  |  | RARB |
|  |  | ASS1 |
|  |  | KEAP1 |
|  |  | PRLR |
|  |  | RRM2B |
|  |  | FGA |
|  |  | TBXAS1 |
|  |  | BLK |
|  |  | NR2F2 |
|  |  | PLD1 |
|  |  | RRM1 |
|  |  | CSNK1D |
|  |  | IL10 |
|  |  | HTR2A |
|  |  | CHAT |
|  |  | EDN1 |
|  |  | IGF1 |
|  |  | IL4R |
|  |  | SLC6A4 |
|  |  | PRKN |
|  |  | REN |
|  |  | POMC |
|  |  | HIF1A |
|  |  | PRNP |
|  |  | SCN1A |
|  |  | GABRG2 |
|  |  | IDS |
|  |  | GFAP |
|  |  | ADRB2 |
|  |  | KCNQ2 |
|  |  | GABRA1 |
|  |  | TBP |
|  |  | ACTA1 |
|  |  | SYT1 |
|  |  | KCNQ3 |
|  |  | CTLA4 |
|  |  | ATP1A2 |
|  |  | TTN |
|  |  | YY1 |
|  |  | TTR |
|  |  | SPTAN1 |
|  |  | GPHN |
|  |  | DHFR |
|  |  | TPO |
|  |  | CD79A |
|  |  | CHRM2 |
|  |  | PAH |
|  |  | MUSK |
|  |  | SDHA |
|  |  | MTHFR |
|  |  | MC1R |
|  |  | ADCY5 |
|  |  | TNNI3 |
|  |  | GALNS |
|  |  | ATP1A3 |
|  |  | DMPK |
|  |  | PSAP |
|  |  | ENO2 |
|  |  | NF1 |
|  |  | TAF1 |
|  |  | ITGAM |
|  |  | TYR |
|  |  | FGF1 |
|  |  | CACNA2D1 |
|  |  | FCGR2A |
|  |  | NOD2 |
|  |  | GRN |
|  |  | PDCD1 |
|  |  | IKBKG |
|  |  | GLB1 |
|  |  | LCAT |
|  |  | OPRM1 |
|  |  | STX1A |
|  |  | MYL2 |
|  |  | HDAC8 |
|  |  | FASLG |
|  |  | ECE1 |
|  |  | KCNB1 |
|  |  | HSPD1 |
|  |  | JUN |
|  |  | ALDH7A1 |
|  |  | MSH6 |
|  |  | SCNN1B |
|  |  | MUC1 |
|  |  | BRD4 |
|  |  | PRKCB |
|  |  | NLRP3 |
|  |  | ODC1 |
|  |  | CYCS |
|  |  | VRK1 |
|  |  | CP |
|  |  | PDE4D |
|  |  | GABRA5 |
|  |  | GLUL |
|  |  | CD28 |
|  |  | MIF |
|  |  | HMGB1 |
|  |  | MYH9 |
|  |  | ABCA1 |
|  |  | HNRNPA1 |
|  |  | SLC12A3 |
|  |  | WT1 |
|  |  | IMPDH2 |
|  |  | CYP19A1 |
|  |  | KNG1 |
|  |  | HCN4 |
|  |  | PMS2 |
|  |  | SCNN1A |
|  |  | NR1H4 |
|  |  | MEF2C |
|  |  | IFNAR1 |
|  |  | CACNA1S |
|  |  | FURIN |
|  |  | MDH2 |
|  |  | KMT2A |
|  |  | PGD |
|  |  | SLC29A1 |
|  |  | TUBB3 |
|  |  | CYBA |
|  |  | CD46 |
|  |  | NF2 |
|  |  | DLG4 |
|  |  | STAT5A |
|  |  | ITGA4 |
|  |  | CHRM3 |
|  |  | SLC2A3 |
|  |  | YWHAG |
|  |  | LIPA |
|  |  | ITPR1 |
|  |  | VWF |
|  |  | LYN |
|  |  | NQO1 |
|  |  | MCL1 |
|  |  | CD44 |
|  |  | PIK3C2A |
|  |  | SLC4A1 |
|  |  | CPT1A |
|  |  | PRKCE |
|  |  | TLR7 |
|  |  | CYP17A1 |
|  |  | KCNJ2 |
|  |  | P2RY12 |
|  |  | WAS |
|  |  | MRE11 |
|  |  | CDK1 |
|  |  | CAV1 |
|  |  | SLC9A3 |
|  |  | TRPA1 |
|  |  | DES |
|  |  | KL |
|  |  | PIK3CG |
|  |  | RHO |
|  |  | ANGPT2 |
|  |  | NBN |
|  |  | F7 |
|  |  | TOP1 |
|  |  | MMP8 |
|  |  | ITGAV |
|  |  | ANGPTL3 |
|  |  | ADAMTS13 |
|  |  | FASN |
|  |  | CAPN3 |
|  |  | PBX1 |
|  |  | SDHB |
|  |  | SLCO1B1 |
|  |  | SCARB1 |
|  |  | AK2 |
|  |  | CDC25C |
|  |  | CACNA1D |
|  |  | PTK2 |
|  |  | HSPA5 |
|  |  | MLH1 |
|  |  | NCAM1 |
|  |  | CDKN1B |
|  |  | ENG |
|  |  | TNFAIP3 |
|  |  | KYNU |
|  |  | FLNA |
|  |  | CAMK2G |
|  |  | DLD |
|  |  | RB1 |
|  |  | RRAS2 |
|  |  | HPRT1 |
|  |  | ITGA3 |
|  |  | L1CAM |
|  |  | ACACA |
|  |  | PCK1 |
|  |  | TPM1 |
|  |  | GATA4 |
|  |  | TRPV1 |
|  |  | ANXA2 |
|  |  | GPI |
|  |  | TLR1 |
|  |  | PLA2G4A |
|  |  | PLCD1 |
|  |  | EIF2AK3 |
|  |  | FUCA1 |
|  |  | CYP1A1 |
|  |  | MAP3K5 |
|  |  | ATP1B1 |
|  |  | IRF7 |
|  |  | ATF6 |
|  |  | ACTG1 |
|  |  | ABCC2 |
|  |  | JUP |
|  |  | PPP2R1A |
|  |  | UBE2I |
|  |  | IFIH1 |
|  |  | ENO3 |
|  |  | MSN |
|  |  | MITF |
|  |  | LEF1 |
|  |  | ADCY1 |
|  |  | TUBB4A |
|  |  | THRA |
|  |  | NCSTN |
|  |  | HSPG2 |
|  |  | KAT2B |
|  |  | CSNK2B |
|  |  | DDX3X |
|  |  | WNT3A |
|  |  | CCND2 |
|  |  | ATP2A1 |
|  |  | KDM6A |
|  |  | CUL3 |
|  |  | DUSP6 |
|  |  | EPHB1 |
|  |  | BMP4 |
|  |  | USP9X |
|  |  | KCNJ1 |
|  |  | NFATC1 |
|  |  | PNLIP |
|  |  | KCNH1 |
|  |  | CTSF |
|  |  | GALK1 |
|  |  | LRP2 |
|  |  | ADORA2B |
|  |  | PPM1D |
|  |  | PGM1 |
|  |  | RRM2 |
|  |  | YAP1 |
|  |  | PI4KA |
|  |  | TAB2 |
|  |  | HLA-DRB1 |
|  |  | IL1B |
|  |  | ADRB1 |
|  |  | INS |
|  |  | GCH1 |
|  |  | PARK7 |
|  |  | GUSB |
|  |  | CSNK1E |
|  |  | TCF4 |
|  |  | ALDH5A1 |
|  |  | ARSB |
|  |  | SMC1A |
|  |  | POLG |
|  |  | NKX2-1 |
|  |  | GLRA1 |
|  |  | RYR1 |
|  |  | TSHR |
|  |  | BRCA2 |
|  |  | MFN2 |
|  |  | CD27 |
|  |  | IGFBP3 |
|  |  | FGF8 |
|  |  | PON1 |
|  |  | SYNGAP1 |
|  |  | PDHA1 |
|  |  | SLC6A1 |
|  |  | FAAH |
|  |  | UNG |
|  |  | KCNK9 |
|  |  | COL3A1 |
|  |  | CD8A |
|  |  | LBR |
|  |  | EIF4G1 |
|  |  | PRPS1 |
|  |  | SLC6A2 |
|  |  | PLCB1 |
|  |  | SMC3 |
|  |  | SOD2 |
|  |  | CACNA1E |
|  |  | ERCC2 |
|  |  | AGER |
|  |  | CTCF |
|  |  | TNK2 |
|  |  | CTSL |
|  |  | TSC1 |
|  |  | TNFRSF11B |
|  |  | F9 |
|  |  | ATP7B |
|  |  | CLU |
|  |  | ITGA7 |
|  |  | P2RX7 |
|  |  | SREBF1 |
|  |  | BACE1 |
|  |  | PLA2G6 |
|  |  | ARSA |
|  |  | GNAI1 |
|  |  | PTPN22 |
|  |  | SCN9A |
|  |  | BAP1 |
|  |  | TNNT2 |
|  |  | IRS1 |
|  |  | ALOX5 |
|  |  | SPTLC2 |
|  |  | HEXB |
|  |  | PAX6 |
|  |  | ABCB4 |
|  |  | GABRA2 |
|  |  | GZMB |
|  |  | CYP11A1 |
|  |  | ADSL |
|  |  | BCL2L1 |
|  |  | PC |
|  |  | FLI1 |
|  |  | MTR |
|  |  | MMP7 |
|  |  | COL1A2 |
|  |  | TUBG1 |
|  |  | PDE2A |
|  |  | HSPA9 |
|  |  | CYSLTR2 |
|  |  | LOX |
|  |  | CARD11 |
|  |  | GNA11 |
|  |  | YWHAB |
|  |  | HADHB |
|  |  | CD81 |
|  |  | CD3D |
|  |  | KCNJ5 |
|  |  | AHR |
|  |  | FSHR |
|  |  | CFH |
|  |  | PLCG1 |
|  |  | GNRHR |
|  |  | TPI1 |
|  |  | CD3E |
|  |  | CD3G |
|  |  | F5 |
|  |  | PCCB |
|  |  | KLK1 |
|  |  | NEDD4 |
|  |  | SLC16A1 |
|  |  | CAPN2 |
|  |  | TGM2 |
|  |  | PDP1 |
|  |  | FPR1 |
|  |  | PLA2G2A |
|  |  | PLIN1 |
|  |  | TNFRSF11A |
|  |  | FLNB |
|  |  | OGG1 |
|  |  | SIRT3 |
|  |  | PLD2 |
|  |  | SLC12A6 |
|  |  | SIGMAR1 |
|  |  | VCL |
|  |  | MVK |
|  |  | FAH |
|  |  | EXT1 |
|  |  | ALDH1A1 |
|  |  | MAG |
|  |  | FOLH1 |
|  |  | ESRRB |
|  |  | DNM1L |
|  |  | ENO1 |
|  |  | NGFR |
|  |  | XDH |
|  |  | TYMP |
|  |  | CAMKK2 |
|  |  | MYCN |
|  |  | CFI |
|  |  | TUBA4A |
|  |  | FGR |
|  |  | UBE2N |
|  |  | TAOK1 |
|  |  | PIN1 |
|  |  | NRP1 |
|  |  | TGFB3 |
|  |  | DCN |
|  |  | ETS1 |
|  |  | ATP6V1B2 |
|  |  | TRPC3 |
|  |  | TFAP2A |
|  |  | TPT1 |
|  |  | MASP2 |
|  |  | IRAK3 |
|  |  | EPHA1 |
|  |  | HSP90B1 |
|  |  | SLC12A1 |
|  |  | IMPA1 |
|  |  | POLA1 |
|  |  | HSD17B3 |
|  |  | CA12 |
|  |  | PTGIR |
|  |  | CSF3R |
|  |  | LIPE |
|  |  | CCR5 |
|  |  | MECP2 |
|  |  | CRP |
|  |  | LEP |
|  |  | MOG |
|  |  | ADIPOQ |
|  |  | CHRNB2 |
|  |  | SLC18A2 |
|  |  | PINK1 |
|  |  | CTSH |
|  |  | UBE3A |
|  |  | APOB |
|  |  | HLA-B |
|  |  | NR3C2 |
|  |  | ADORA2A |
|  |  | HTR1A |
|  |  | IL4 |
|  |  | MYH7 |
|  |  | IL2 |
|  |  | SLC9A6 |
|  |  | PRKAR1B |
|  |  | KCNA2 |
|  |  | DCTN1 |
|  |  | IDO1 |
|  |  | IL1R1 |
|  |  | MC4R |
|  |  | GABRD |
|  |  | NR4A2 |
|  |  | GRM7 |
|  |  | CNR1 |
|  |  | GABBR1 |
|  |  | AQP4 |
|  |  | EHMT1 |
|  |  | HTR3A |
|  |  | HLA-A |
|  |  | WFS1 |
|  |  | PPT1 |
|  |  | CYP2D6 |
|  |  | RORB |
|  |  | FGF2 |
|  |  | SLC25A13 |
|  |  | ADORA1 |
|  |  | MBL2 |
|  |  | MYBPC3 |
|  |  | EEF1A2 |
|  |  | TARDBP |
|  |  | PLCB4 |
|  |  | BMP2 |
|  |  | DHCR7 |
|  |  | GHR |
|  |  | TGIF1 |
|  |  | FBN1 |
|  |  | GLRB |
|  |  | HTRA2 |
|  |  | CACNB4 |
|  |  | OXTR |
|  |  | ACVR2A |
|  |  | QDPR |
|  |  | SMARCB1 |
|  |  | PNKP |
|  |  | SCN4A |
|  |  | SUCLG1 |
|  |  | GHSR |
|  |  | AQP2 |
|  |  | IL10RA |
|  |  | PAFAH1B1 |
|  |  | CD38 |
|  |  | ST3GAL3 |
|  |  | HMGCR |
|  |  | LAMB2 |
|  |  | CSTB |
|  |  | GOT2 |
|  |  | RUNX2 |
|  |  | CYP2C9 |
|  |  | KCNE1 |
|  |  | FOXP3 |
|  |  | DRD5 |
|  |  | GRM5 |
|  |  | TPM2 |
|  |  | CTSA |
|  |  | SLC25A12 |
|  |  | TPM3 |
|  |  | TLR9 |
|  |  | SCN3B |
|  |  | MYOD1 |
|  |  | PNPO |
|  |  | NLGN1 |
|  |  | SEMA3A |
|  |  | HYAL1 |
|  |  | HSPA1A |
|  |  | RYR2 |
|  |  | CR2 |
|  |  | KCNJ11 |
|  |  | ITGAL |
|  |  | IRF5 |
|  |  | NCF1 |
|  |  | GNB3 |
|  |  | MC2R |
|  |  | UCP2 |
|  |  | CACNB2 |
|  |  | SCN2B |
|  |  | SLC22A5 |
|  |  | TUBB1 |
|  |  | CD14 |
|  |  | AFP |
|  |  | STUB1 |
|  |  | KLK3 |
|  |  | KITLG |
|  |  | PTGDR |
|  |  | NR1H2 |
|  |  | PPARGC1A |
|  |  | ABCB11 |
|  |  | OGDH |
|  |  | MST1 |
|  |  | FCGR3A |
|  |  | SCARB2 |
|  |  | ABAT |
|  |  | KRT14 |
|  |  | SERPINI1 |
|  |  | GALNT2 |
|  |  | SPP1 |
|  |  | NPHS1 |
|  |  | TRAF3 |
|  |  | ORC1 |
|  |  | TNFRSF13B |
|  |  | CD59 |
|  |  | FOXO3 |
|  |  | GJB1 |
|  |  | YWHAH |
|  |  | LRP1 |
|  |  | F13A1 |
|  |  | FTO |
|  |  | EHMT2 |
|  |  | HTR1D |
|  |  | HNF1A |
|  |  | CASP9 |
|  |  | EPB41 |
|  |  | IRF1 |
|  |  | CYP24A1 |
|  |  | FECH |
|  |  | SORL1 |
|  |  | HADHA |
|  |  | CYP27A1 |
|  |  | GFPT1 |
|  |  | ADRA1B |
|  |  | SETD2 |
|  |  | ANXA5 |
|  |  | YWHAZ |
|  |  | VDAC1 |
|  |  | GLDC |
|  |  | TGFA |
|  |  | PFN1 |
|  |  | COL5A1 |
|  |  | GRIK1 |
|  |  | GRIN2C |
|  |  | SUCLA2 |
|  |  | BIN1 |
|  |  | FH |
|  |  | NTF4 |
|  |  | USP15 |
|  |  | ALDH18A1 |
|  |  | DLAT |
|  |  | PAX2 |
|  |  | FYN |
|  |  | PMM2 |
|  |  | GPX4 |
|  |  | PTGS1 |
|  |  | NR1H3 |
|  |  | ATP6V1A |
|  |  | ADRA1A |
|  |  | GABRA3 |
|  |  | CALM1 |
|  |  | COMP |
|  |  | APAF1 |
|  |  | CETP |
|  |  | AGL |
|  |  | FDPS |
|  |  | SLC6A8 |
|  |  | ACSL1 |
|  |  | STAT4 |
|  |  | CSNK2A2 |
|  |  | CRYAB |
|  |  | TGFBI |
|  |  | GPC3 |
|  |  | MDH1 |
|  |  | ACP5 |
|  |  | PCK2 |
|  |  | GRM3 |
|  |  | CA4 |
|  |  | DAG1 |
|  |  | PCCA |
|  |  | LYZ |
|  |  | SHMT2 |
|  |  | REL |
|  |  | ISG15 |
|  |  | ABCD1 |
|  |  | AVPR2 |
|  |  | LIG4 |
|  |  | SGPL1 |
|  |  | CHD7 |
|  |  | UQCRC2 |
|  |  | IL1R2 |
|  |  | PRKACG |
|  |  | SSTR2 |
|  |  | AVPR1A |
|  |  | TLR5 |
|  |  | NOTCH4 |
|  |  | KDM5C |
|  |  | TCF3 |
|  |  | ACACB |
|  |  | MAX |
|  |  | TDO2 |
|  |  | TRAF6 |
|  |  | PRDX2 |
|  |  | CLTC |
|  |  | F2R |
|  |  | PDE10A |
|  |  | HEXA |
|  |  | CNTN2 |
|  |  | NR0B1 |
|  |  | FPR2 |
|  |  | MSTN |
|  |  | SPRY2 |
|  |  | ACTN4 |
|  |  | GNAI2 |
|  |  | RPSA |
|  |  | ACTN2 |
|  |  | ABCC8 |
|  |  | WNT1 |
|  |  | ATRX |
|  |  | SLC4A4 |
|  |  | TCF12 |
|  |  | HSF1 |
|  |  | ACAN |
|  |  | TMPO |
|  |  | ACTA2 |
|  |  | KCNC1 |
|  |  | FGF10 |
|  |  | STS |
|  |  | DDB2 |
|  |  | KRT8 |
|  |  | FMO3 |
|  |  | RASA1 |
|  |  | AQP5 |
|  |  | PIK3C2B |
|  |  | IL5RA |
|  |  | PLCB2 |
|  |  | TPMT |
|  |  | VIPR1 |
|  |  | HCFC1 |
|  |  | CLDN1 |
|  |  | SERPINF1 |
|  |  | KCNA5 |
|  |  | IL17RA |
|  |  | CDK12 |
|  |  | KCNC3 |
|  |  | FGB |
|  |  | LNPEP |
|  |  | MMP12 |
|  |  | THBD |
|  |  | CASP2 |
|  |  | PKLR |
|  |  | EIF4EBP1 |
|  |  | EPHA3 |
|  |  | LHCGR |
|  |  | PIP4K2A |
|  |  | APRT |
|  |  | PYGM |
|  |  | RXRB |
|  |  | SLC19A3 |
|  |  | EZR |
|  |  | ROS1 |
|  |  | PSMB9 |
|  |  | ITGAX |
|  |  | GALT |
|  |  | TCF7L2 |
|  |  | CCND3 |
|  |  | LIG1 |
|  |  | CTNNA1 |
|  |  | PDXK |
|  |  | GATA6 |
|  |  | WNT3 |
|  |  | DDR1 |
|  |  | CCN2 |
|  |  | GRHPR |
|  |  | RIT1 |
|  |  | PSAT1 |
|  |  | AKR1C3 |
|  |  | GRK6 |
|  |  | GRM6 |
|  |  | IGFBP7 |
|  |  | CDH11 |
|  |  | LTA4H |
|  |  | LPAR1 |
|  |  | OPRL1 |
|  |  | HDAC7 |
|  |  | COL4A3 |
|  |  | WRN |
|  |  | ITGA2 |
|  |  | XPC |
|  |  | FER |
|  |  | ERCC3 |
|  |  | TUBB2A |
|  |  | CARM1 |
|  |  | CTBP1 |
|  |  | AKR1B1 |
|  |  | CBFB |
|  |  | ITPR3 |
|  |  | GLP1R |
|  |  | FBLN5 |
|  |  | FANCD2 |
|  |  | ABCC3 |
|  |  | ARHGEF2 |
|  |  | GATA1 |
|  |  | SLC40A1 |
|  |  | TALDO1 |
|  |  | CHN1 |
|  |  | EIF4A1 |
|  |  | NT5C2 |
|  |  | PPP2R1B |
|  |  | RPS6KA5 |
|  |  | SERPIND1 |
|  |  | DCC |
|  |  | CPE |
|  |  | ENPEP |
|  |  | PML |
|  |  | RPS6KA2 |
|  |  | NOG |
|  |  | KLKB1 |
|  |  | ADCY3 |
|  |  | POLD1 |
|  |  | AK1 |
|  |  | PTGER3 |
|  |  | ROR1 |
|  |  | CCR2 |
|  |  | CCL11 |
|  |  | HCRTR2 |
|  |  | ATXN3 |
|  |  | CYP2B6 |
|  |  | ABCC9 |
|  |  | VCAM1 |
|  |  | IDUA |
|  |  | ACHE |
|  |  | HTR2C |
|  |  | PTGDS |
|  |  | TSPO |
|  |  | CSF2 |
|  |  | SCN1B |
|  |  | CCR3 |
|  |  | IL1A |
|  |  | HERC2 |
|  |  | BCL6 |
|  |  | SELP |
|  |  | TAT |
|  |  | STAG2 |
|  |  | HRH1 |
|  |  | HP |
|  |  | MTNR1B |
|  |  | SLC25A1 |
|  |  | SOX2 |
|  |  | DMD |
|  |  | GNAO1 |
|  |  | HLA-DPB1 |
|  |  | CLCN2 |
|  |  | NEFL |
|  |  | IL12RB1 |
|  |  | SYNJ1 |
|  |  | DRD4 |
|  |  | NPPA |
|  |  | TFAP2B |
|  |  | PTS |
|  |  | IL5 |
|  |  | CALM2 |
|  |  | CHRM1 |
|  |  | SIN3A |
|  |  | SPTBN1 |
|  |  | LTF |
|  |  | SYP |
|  |  | OTX2 |
|  |  | CCR1 |
|  |  | SLC17A5 |
|  |  | TWIST1 |
|  |  | WWOX |
|  |  | ALDH4A1 |
|  |  | IL3 |
|  |  | HBB |
|  |  | CDKN1C |
|  |  | NEFH |
|  |  | GNAI3 |
|  |  | DDB1 |
|  |  | CHRNA7 |
|  |  | HUWE1 |
|  |  | SKI |
|  |  | KAT6A |
|  |  | COL6A1 |
|  |  | PRTN3 |
|  |  | GJB2 |
|  |  | CYP1A2 |
|  |  | ERCC4 |
|  |  | NONO |
|  |  | TPH1 |
|  |  | OPRK1 |
|  |  | FBXW11 |
|  |  | TXN |
|  |  | PHKA2 |
|  |  | CD34 |
|  |  | FTL |
|  |  | IL12B |
|  |  | TIMP1 |
|  |  | HNMT |
|  |  | FXN |
|  |  | IGF2R |
|  |  | CHRNA3 |
|  |  | MARS1 |
|  |  | SMARCAL1 |
|  |  | ADAR |
|  |  | HMGCL |
|  |  | AGA |
|  |  | APOA2 |
|  |  | GPX1 |
|  |  | CD274 |
|  |  | BMP6 |
|  |  | TCIRG1 |
|  |  | POU5F1 |
|  |  | LARS2 |
|  |  | TKT |
|  |  | RAD21 |
|  |  | SLC5A5 |
|  |  | TNFSF13B |
|  |  | ITGB7 |
|  |  | HNRNPK |
|  |  | ARID1A |
|  |  | ACADM |
|  |  | CORIN |
|  |  | RBP4 |
|  |  | PTH |
|  |  | PABPN1 |
|  |  | YWHAQ |
|  |  | SP1 |
|  |  | ADRA2A |
|  |  | ATP13A2 |
|  |  | DOCK8 |
|  |  | COL6A2 |
|  |  | NEU1 |
|  |  | EIF5A |
|  |  | PLCE1 |
|  |  | RBL2 |
|  |  | NAA10 |
|  |  | CHRNE |
|  |  | IRF8 |
|  |  | IL21 |
|  |  | CCKAR |
|  |  | LGALS3 |
|  |  | CCNK |
|  |  | CTSG |
|  |  | SOX5 |
|  |  | DAO |
|  |  | HLA-DRA |
|  |  | CRAT |
|  |  | CDH5 |
|  |  | FBXW7 |
|  |  | PDE4A |
|  |  | CGA |
|  |  | PAX5 |
|  |  | GLRA2 |
|  |  | MSR1 |
|  |  | SERPINA6 |
|  |  | SI |
|  |  | DHODH |
|  |  | PTPRD |
|  |  | GRID2 |
|  |  | PPP2R2B |
|  |  | KCNJ10 |
|  |  | VAPB |
|  |  | CHRM4 |
|  |  | LARS1 |
|  |  | PANK2 |
|  |  | CFL1 |
|  |  | HRH4 |
|  |  | CHRM5 |
|  |  | CHRNA5 |
|  |  | COASY |
|  |  | MYH6 |
|  |  | PRODH |
|  |  | FANCL |
|  |  | RAB11A |
|  |  | KLK6 |
|  |  | CYP27B1 |
|  |  | ABCA7 |
|  |  | HSD3B2 |
|  |  | BID |
|  |  | GARS1 |
|  |  | TLR6 |
|  |  | AFG3L2 |
|  |  | PLAUR |
|  |  | CANX |
|  |  | SMARCE1 |
|  |  | BECN1 |
|  |  | ALDH3A2 |
|  |  | LIPC |
|  |  | ALDH6A1 |
|  |  | EFEMP1 |
|  |  | TOR1A |
|  |  | AKR1D1 |
|  |  | AGXT |
|  |  | BIRC5 |
|  |  | CISH |
|  |  | ATP6AP2 |
|  |  | GNB1 |
|  |  | LCN2 |
|  |  | HSD11B2 |
|  |  | CCR4 |
|  |  | SNTA1 |
|  |  | APEX1 |
|  |  | HNRNPA2B1 |
|  |  | SLC2A4 |
|  |  | KRT5 |
|  |  | MPZ |
|  |  | PTHLH |
|  |  | OTC |
|  |  | TET2 |
|  |  | PRKAB2 |
|  |  | MAN2B1 |
|  |  | HAVCR2 |
|  |  | PCBD1 |
|  |  | BAK1 |
|  |  | NEDD4L |
|  |  | NDUFS3 |
|  |  | RPE65 |
|  |  | F3 |
|  |  | XBP1 |
|  |  | CYP2E1 |
|  |  | OSMR |
|  |  | MMAB |
|  |  | CCNA2 |
|  |  | MCM5 |
|  |  | CHRNB4 |
|  |  | GCLC |
|  |  | ICOS |
|  |  | UBE2L3 |
|  |  | CRYAA |
|  |  | FLNC |
|  |  | NDUFV1 |
|  |  | ALAS2 |
|  |  | TOP2B |
|  |  | MUTYH |
|  |  | PLOD1 |
|  |  | P2RY2 |
|  |  | PTK7 |
|  |  | GLO1 |
|  |  | PKD1 |
|  |  | ATF4 |
|  |  | EGR1 |
|  |  | ARG2 |
|  |  | ANG |
|  |  | PTPRM |
|  |  | STRADA |
|  |  | MAD1L1 |
|  |  | TUFM |
|  |  | PSPH |
|  |  | APOC3 |
|  |  | CS |
|  |  | LONP1 |
|  |  | PDHX |
|  |  | GPC6 |
|  |  | ETFA |
|  |  | SCP2 |
|  |  | ABCB6 |
|  |  | SCO1 |
|  |  | SIRT5 |
|  |  | TEC |
|  |  | DPYSL2 |
|  |  | HABP2 |
|  |  | PAK5 |
|  |  | FA2H |
|  |  | CBLB |
|  |  | LMAN1 |
|  |  | COL6A3 |
|  |  | ALG1 |
|  |  | ACADVL |
|  |  | PEPD |
|  |  | GRM2 |
|  |  | HTRA1 |
|  |  | THBS1 |
|  |  | PDE5A |
|  |  | ATIC |
|  |  | ALOX15 |
|  |  | SHMT1 |
|  |  | CELF2 |
|  |  | XRCC6 |
|  |  | BTD |
|  |  | SOX10 |
|  |  | DCLK1 |
|  |  | PPP2R5D |
|  |  | FUT2 |
|  |  | HFE |
|  |  | KLK4 |
|  |  | CTSS |
|  |  | NAGA |
|  |  | AMH |
|  |  | CLCN7 |
|  |  | OGT |
|  |  | NDRG1 |
|  |  | ADCY9 |
|  |  | TNFRSF10A |
|  |  | TNKS |
|  |  | RTN4 |
|  |  | MTMR2 |
|  |  | GYS2 |
|  |  | EMD |
|  |  | ATG5 |
|  |  | TNIK |
|  |  | SOX9 |
|  |  | PXDN |
|  |  | ADCYAP1R1 |
|  |  | EPS8 |
|  |  | TUBA8 |
|  |  | DDIT3 |
|  |  | GPNMB |
|  |  | KLF4 |
|  |  | SLC19A2 |
|  |  | DPAGT1 |
|  |  | TGM1 |
|  |  | SLC39A8 |
|  |  | PITX2 |
|  |  | SOCS1 |
|  |  | HPSE |
|  |  | PSENEN |
|  |  | SIAH1 |
|  |  | ADRA1D |
|  |  | PVR |
|  |  | CKM |
|  |  | ENPP2 |
|  |  | IL23R |
|  |  | BMP7 |
|  |  | CASQ2 |
|  |  | BTRC |
|  |  | ADCY2 |
|  |  | ARRB2 |
|  |  | GSTO1 |
|  |  | AKR1A1 |
|  |  | MAP2K7 |
|  |  | PPARD |
|  |  | HSPB8 |
|  |  | GABRA6 |
|  |  | GAMT |
|  |  | EFNB1 |
|  |  | ADAM12 |
|  |  | HINT1 |
|  |  | XPA |
|  |  | ALOX12 |
|  |  | ANTXR1 |
|  |  | ANGPT1 |
|  |  | NCOA2 |
|  |  | ALDH1A3 |
|  |  | GRK3 |
|  |  | POMGNT1 |
|  |  | TK1 |
|  |  | FGF3 |
|  |  | DCT |
|  |  | FKBP4 |
|  |  | LIG3 |
|  |  | MYH11 |
|  |  | DSG2 |
|  |  | ASL |
|  |  | PTPRN2 |
|  |  | GNAT1 |
|  |  | NRP2 |
|  |  | ADORA3 |
|  |  | TLK2 |
|  |  | TUBB4B |
|  |  | SOS2 |
|  |  | AP2M1 |
|  |  | ATP7A |
|  |  | CCT5 |
|  |  | TRPM8 |
|  |  | MVD |
|  |  | NSF |
|  |  | HMOX2 |
|  |  | CACNA1F |
|  |  | TSPAN7 |
|  |  | TBX21 |
|  |  | PSMB1 |
|  |  | ILK |
|  |  | CTNND1 |
|  |  | KAT6B |
|  |  | PDX1 |
|  |  | CAMK4 |
|  |  | OCRL |
|  |  | MDM4 |
|  |  | NFATC2 |
|  |  | ABCC4 |
|  |  | ATP6V1B1 |
|  |  | ERCC1 |
|  |  | PPP2CB |
|  |  | LAMB3 |
|  |  | CUBN |
|  |  | SEMA3E |
|  |  | SEMA4A |
|  |  | BDKRB2 |
|  |  | MYL3 |
|  |  | TFR2 |
|  |  | TNFRSF13C |
|  |  | WDR5 |
|  |  | ZIC3 |
|  |  | MYH10 |
|  |  | GK |
|  |  | KDM6B |
|  |  | TBL1XR1 |
|  |  | TRRAP |
|  |  | ULK1 |
|  |  | UROD |
|  |  | DBI |
|  |  | HPD |
|  |  | SLC39A14 |
|  |  | PTGFR |
|  |  | PTPRS |
|  |  | RALBP1 |
|  |  | CRY1 |
|  |  | CXCL12 |
|  |  | PHOX2B |
|  |  | CDKL5 |
|  |  | CLOCK |
|  |  | CCL5 |
|  |  | HLA-C |
|  |  | CXCL8 |
|  |  | P2RY11 |
|  |  | SNRPN |
|  |  | S100B |
|  |  | NPY |
|  |  | GNS |
|  |  | HTT |
|  |  | IL7 |
|  |  | CHI3L1 |
|  |  | DRD3 |
|  |  | CXCL10 |
|  |  | FMR1 |
|  |  | IL18 |
|  |  | FLII |
|  |  | GAL |
|  |  | MAOB |
|  |  | FXR1 |
|  |  | TPP1 |
|  |  | PAX8 |
|  |  | SATB2 |
|  |  | APOL1 |
|  |  | OSM |
|  |  | ATN1 |
|  |  | CLCN1 |
|  |  | SLC18A3 |
|  |  | CHKA |
|  |  | TG |
|  |  | SMS |
|  |  | IFNA2 |
|  |  | AVP |
|  |  | TREM2 |
|  |  | DRD1 |
|  |  | DISC1 |
|  |  | IL12A |
|  |  | RELN |
|  |  | VIP |
|  |  | TACR1 |
|  |  | CYP2C19 |
|  |  | DUOX2 |
|  |  | ARNT2 |
|  |  | POLR2A |
|  |  | NGLY1 |
|  |  | RNASEL |
|  |  | SEPSECS |
|  |  | MAP2 |
|  |  | MBP |
|  |  | PLN |
|  |  | IGFBP1 |
|  |  | CCR7 |
|  |  | GALC |
|  |  | SATB1 |
|  |  | EPRS1 |
|  |  | EYA1 |
|  |  | CXCR3 |
|  |  | BSCL2 |
|  |  | CRHR1 |
|  |  | PURA |
|  |  | NSDHL |
|  |  | DKK1 |
|  |  | IRF4 |
|  |  | CR1 |
|  |  | TXNRD2 |
|  |  | VIPR2 |
|  |  | MLXIPL |
|  |  | LGI1 |
|  |  | CDH23 |
|  |  | FUS |
|  |  | REV3L |
|  |  | PRMT7 |
|  |  | CNR2 |
|  |  | NR1I2 |
|  |  | TFE3 |
|  |  | MEN1 |
|  |  | STAR |
|  |  | GJA8 |
|  |  | CST3 |
|  |  | ARID1B |
|  |  | GJA5 |
|  |  | CYP3A5 |
|  |  | CHMP2B |
|  |  | SLC12A4 |
|  |  | CYP21A2 |
|  |  | MAF |
|  |  | GAD2 |
|  |  | SMARCC2 |
|  |  | SFTPD |
|  |  | CSF1 |
|  |  | EIF3F |
|  |  | UGP2 |
|  |  | CPT1B |
|  |  | ARL3 |
|  |  | PITX1 |
|  |  | CD80 |
|  |  | FHIT |
|  |  | PDYN |
|  |  | TJP1 |
|  |  | MAP2K5 |
|  |  | RFC2 |
|  |  | CD86 |
|  |  | LAMP2 |
|  |  | CPOX |
|  |  | G6PC1 |
|  |  | PKP2 |
|  |  | DYNC1H1 |
|  |  | FARS2 |
|  |  | SRD5A1 |
|  |  | HMBS |
|  |  | HTR3B |
|  |  | NKX2-5 |
|  |  | APOH |
|  |  | IREB2 |
|  |  | DCX |
|  |  | C5AR1 |
|  |  | IGFBP2 |
|  |  | ADM |
|  |  | GPD1 |
|  |  | ACO1 |
|  |  | KMT2C |
|  |  | NR1I3 |
|  |  | APBB1 |
|  |  | MGAM |
|  |  | DPM1 |
|  |  | MADD |
|  |  | COL17A1 |
|  |  | FOXP2 |
|  |  | SPG7 |
|  |  | MAP1B |
|  |  | POMT1 |
|  |  | NMT1 |
|  |  | SFTPA1 |
|  |  | LMX1B |
|  |  | CX3CL1 |
|  |  | DGUOK |
|  |  | EXT2 |
|  |  | KRT19 |
|  |  | LBP |
|  |  | CD163 |
|  |  | BSG |
|  |  | COL4A5 |
|  |  | FIG4 |
|  |  | CDH13 |
|  |  | PGF |
|  |  | ALPP |
|  |  | APOA5 |
|  |  | SLC8A1 |
|  |  | KIF1B |
|  |  | SYN1 |
|  |  | GMPPB |
|  |  | TDP2 |
|  |  | LAMA2 |
|  |  | POMT2 |
|  |  | NDUFS1 |
|  |  | NTF3 |
|  |  | KIF1A |
|  |  | ELP1 |
|  |  | OPA1 |
|  |  | EWSR1 |
|  |  | PRDX5 |
|  |  | NR2F1 |
|  |  | ACP1 |
|  |  | PPIF |
|  |  | SLC29A2 |
|  |  | PSMC3 |
|  |  | CHIT1 |
|  |  | GYPA |
|  |  | BCS1L |
|  |  | SOST |
|  |  | GFM1 |
|  |  | CD47 |
|  |  | TRAP1 |
|  |  | COL4A4 |
|  |  | LTBP2 |
|  |  | USP14 |
|  |  | DNAJB1 |
|  |  | ME2 |
|  |  | SUOX |
|  |  | NTHL1 |
|  |  | KDM4A |
|  |  | EGR2 |
|  |  | ADCY10 |
|  |  | CIITA |
|  |  | ALDH1B1 |
|  |  | VAMP2 |
|  |  | RNF2 |
|  |  | CARD9 |
|  |  | AMT |
|  |  | NLGN3 |
|  |  | SORT1 |
|  |  | NQO2 |
|  |  | SDHD |
|  |  | UBB |
|  |  | KCNN2 |
|  |  | MEFV |
|  |  | ACAA2 |
|  |  | CD151 |
|  |  | XRCC1 |
|  |  | SGCD |
|  |  | NCL |
|  |  | CCKBR |
|  |  | ID2 |
|  |  | TXNRD1 |
|  |  | NOP56 |
|  |  | VTN |
|  |  | SLC13A5 |
|  |  | AMACR |
|  |  | PICALM |
|  |  | ADH1B |
|  |  | MKI67 |
|  |  | SELPLG |
|  |  | PCNT |
|  |  | SLC25A15 |
|  |  | EXTL3 |
|  |  | HIPK2 |
|  |  | PPARA |
|  |  | NDUFB9 |
|  |  | C1QBP |
|  |  | FOXP1 |
|  |  | CD2 |
|  |  | ABCG5 |
|  |  | NDUFS2 |
|  |  | RELB |
|  |  | PPOX |
|  |  | HTR4 |
|  |  | ST3GAL1 |
|  |  | NPC1L1 |
|  |  | PDE4B |
|  |  | SLC7A11 |
|  |  | TUBA1B |
|  |  | DPEP1 |
|  |  | ICAM2 |
|  |  | ACAD8 |
|  |  | GC |
|  |  | FDXR |
|  |  | SLC5A6 |
|  |  | SV2A |
|  |  | PLA2G1B |
|  |  | LETM1 |
|  |  | TJP2 |
|  |  | TRPS1 |
|  |  | XRCC5 |
|  |  | DLK1 |
|  |  | AARS1 |
|  |  | CHD4 |
|  |  | ADARB1 |
|  |  | SLC10A1 |
|  |  | ADIPOR1 |
|  |  | DROSHA |
|  |  | SLC16A2 |
|  |  | RAB5A |
|  |  | APOC2 |
|  |  | DIAPH3 |
|  |  | ACTC1 |
|  |  | POLR2B |
|  |  | EPS15 |
|  |  | GDI1 |
|  |  | CFL2 |
|  |  | UGT1A9 |
|  |  | NCOR1 |
|  |  | MMP20 |
|  |  | BRD2 |
|  |  | MPDZ |
|  |  | TRAF2 |
|  |  | PTPN13 |
|  |  | IFNGR2 |
|  |  | AHSG |
|  |  | SPTA1 |
|  |  | ITGA1 |
|  |  | ALG8 |
|  |  | SLC13A3 |
|  |  | F2RL1 |
|  |  | EBP |
|  |  | TUBB2B |
|  |  | TNKS2 |
|  |  | ISL1 |
|  |  | SKP1 |
|  |  | CLIP1 |
|  |  | NUP155 |
|  |  | ARRB1 |
|  |  | AP1B1 |
|  |  | PSMD14 |
|  |  | RBBP8 |
|  |  | PPP3CB |
|  |  | NNMT |
|  |  | DUSP3 |
|  |  | CYB5A |
|  |  | MED12 |
|  |  | USP5 |
|  |  | ROBO4 |
|  |  | TNFRSF4 |
|  |  | CKB |
|  |  | MYO1E |
|  |  | NLRP12 |
|  |  | PPP1R12A |
|  |  | HARS1 |
|  |  | GDAP1 |
|  |  | TRIM24 |
|  |  | CMA1 |
|  |  | SLC6A19 |
|  |  | AMBP |
|  |  | NAT1 |
|  |  | SFTPB |
|  |  | PTGDR2 |
|  |  | NR5A2 |
|  |  | PTPN3 |
|  |  | ALOX5AP |
|  |  | IL12RB2 |
|  |  | PLK3 |
|  |  | MAPKAP1 |
|  |  | POLR1C |
|  |  | ANGPTL4 |
|  |  | DHX38 |
|  |  | FADS2 |
|  |  | GGPS1 |
|  |  | GRB14 |
|  |  | PCSK7 |
|  |  | POLL |
|  |  | SLC22A1 |
|  |  | ALS2 |
|  |  | FUT8 |
|  |  | LTC4S |
|  |  | CORO1A |
|  |  | GPC4 |
|  |  | CNTFR |
|  |  | SQLE |
|  |  | KCNB2 |
|  |  | PCSK2 |
|  |  | PTPRN |
|  |  | EOMES |
|  |  | IL1RAPL1 |
|  |  | TAPBP |
|  |  | AGTR2 |
|  |  | CADM1 |
|  |  | PGRMC1 |
|  |  | BGN |
|  |  | NUP107 |
|  |  | SERPINA5 |
|  |  | SLCO2A1 |
|  |  | PDE3B |
|  |  | PTPRU |
|  |  | EIF2AK1 |
|  |  | ADCY8 |
|  |  | CFD |
|  |  | GART |
|  |  | PDIA3 |
|  |  | APTX |
|  |  | CSTA |
|  |  | MGLL |
|  |  | RORC |
|  |  | CRBN |
|  |  | FZR1 |
|  |  | UFD1 |
|  |  | AOX1 |
|  |  | DOCK1 |
|  |  | E2F4 |
|  |  | GIPR |
|  |  | RAD23B |
|  |  | SF3B4 |
|  |  | TNNI2 |
|  |  | FGD4 |
|  |  | BTC |
|  |  | HJV |
|  |  | KIF22 |
|  |  | NANS |
|  |  | RSPO1 |
|  |  | SERPINB2 |
|  |  | SLC2A10 |
|  |  | SLC7A5 |
|  |  | TFG |
|  |  | ADGRG6 |
|  |  | CRADD |
|  |  | GAB1 |
|  |  | PARP4 |
|  |  | EIF3A |
|  |  | CHST3 |
|  |  | SMAD7 |
|  |  | VARS1 |
|  |  | PER2 |
|  |  | CRH |
|  |  | CX3CR1 |
|  |  | CD209 |
|  |  | AICDA |
|  |  | KCNT1 |
|  |  | CXCR1 |
|  |  | NPPB |
|  |  | PRL |
|  |  | NAGLU |
|  |  | MTNR1A |
|  |  | RETN |
|  |  | IL13 |
|  |  | OCA2 |
|  |  | GH1 |
|  |  | ATXN2 |
|  |  | ADNP |
|  |  | SNCB |
|  |  | IL15 |
|  |  | SNCAIP |
|  |  | ASXL1 |
|  |  | VAMP1 |
|  |  | ARX |
|  |  | PCDH19 |
|  |  | GLUD2 |
|  |  | IL17A |
|  |  | ORC4 |
|  |  | ELN |
|  |  | HRH3 |
|  |  | HIBCH |
|  |  | IFNB1 |
|  |  | CAV3 |
|  |  | SELL |
|  |  | SELE |
|  |  | SOX3 |
|  |  | ZIC2 |
|  |  | SLC5A7 |
|  |  | SIX3 |
|  |  | DNAJC6 |
|  |  | NDUFS4 |
|  |  | CDK13 |
|  |  | SERPINA3 |
|  |  | CLN3 |
|  |  | GRB10 |
|  |  | CYP51A1 |
|  |  | EPM2A |
|  |  | FBXO7 |
|  |  | NPC2 |
|  |  | HLA-G |
|  |  | IL18R1 |
|  |  | LIAS |
|  |  | CCR6 |
|  |  | SETD1A |
|  |  | CALCA |
|  |  | CXCL1 |
|  |  | EPX |
|  |  | DHPS |
|  |  | SLC26A4 |
|  |  | CEP57 |
|  |  | C4A |
|  |  | SARDH |
|  |  | HSPA4 |
|  |  | NFIX |
|  |  | ARSG |
|  |  | PODXL |
|  |  | FCER2 |
|  |  | MYO7A |
|  |  | HLA-DQA1 |
|  |  | PRPH |
|  |  | NRXN3 |
|  |  | ERCC8 |
|  |  | SLC28A1 |
|  |  | GPD1L |
|  |  | HTR1B |
|  |  | ADRB3 |
|  |  | PMP22 |
|  |  | CIC |
|  |  | CCL7 |
|  |  | GPLD1 |
|  |  | HMGA2 |
|  |  | USH1C |
|  |  | ATXN1 |
|  |  | HAMP |
|  |  | PIGK |
|  |  | FCGR3B |
|  |  | FGF12 |
|  |  | TNFRSF8 |
|  |  | PTX3 |
|  |  | KCNE2 |
|  |  | UBQLN2 |
|  |  | RARS2 |
|  |  | SULT2A1 |
|  |  | SDC1 |
|  |  | TNXB |
|  |  | DOCK6 |
|  |  | SLC25A11 |
|  |  | ABCB7 |
|  |  | LAMP1 |
|  |  | OPTN |
|  |  | AGO2 |
|  |  | LAMA5 |
|  |  | AFF2 |
|  |  | TYROBP |
|  |  | POLI |
|  |  | MTM1 |
|  |  | AIRE |
|  |  | H2AX |
|  |  | M6PR |
|  |  | CYSLTR1 |
|  |  | PNPT1 |
|  |  | GDF15 |
|  |  | LIF |
|  |  | TICAM1 |
|  |  | CTNND2 |
|  |  | DPP10 |
|  |  | CDT1 |
|  |  | HRH2 |
|  |  | APCS |
|  |  | CLCNKB |
|  |  | UBC |
|  |  | OPCML |
|  |  | SUFU |
|  |  | PON2 |
|  |  | HTR2B |
|  |  | HSD3B1 |
|  |  | CNBP |
|  |  | OPHN1 |
|  |  | EEF1A1 |
|  |  | DYSF |
|  |  | FGF4 |
|  |  | CHGA |
|  |  | HAVCR1 |
|  |  | RBX1 |
|  |  | ANXA11 |
|  |  | NEK1 |
|  |  | ADAMTSL1 |
|  |  | SREBF2 |
|  |  | CEBPB |
|  |  | DLG2 |
|  |  | FABP4 |
|  |  | MARS2 |
|  |  | TFAM |
|  |  | HNRNPU |
|  |  | CD1D |
|  |  | TIRAP |
|  |  | ICAM3 |
|  |  | VDAC3 |
|  |  | GAP43 |
|  |  | MCCC1 |
|  |  | PAPPA |
|  |  | PNMT |
|  |  | RPL10 |
|  |  | GYPC |
|  |  | PLXND1 |
|  |  | USP4 |
|  |  | UGT2B7 |
|  |  | S100A9 |
|  |  | SOCS3 |
|  |  | NDUFV2 |
|  |  | SLC25A10 |
|  |  | FGF7 |
|  |  | MBD4 |
|  |  | CBX5 |
|  |  | AVPR1B |
|  |  | MLYCD |
|  |  | COQ7 |
|  |  | COL7A1 |
|  |  | DAB1 |
|  |  | COQ8A |
|  |  | DNAH11 |
|  |  | GPAM |
|  |  | FABP7 |
|  |  | PAM |
|  |  | TRIM32 |
|  |  | UCP1 |
|  |  | EXOSC3 |
|  |  | UBE2D2 |
|  |  | KPNB1 |
|  |  | FABP3 |
|  |  | NDUFS6 |
|  |  | COX5A |
|  |  | LTBP4 |
|  |  | AMPH |
|  |  | MANBA |
|  |  | SPRED1 |
|  |  | MOGS |
|  |  | MCOLN1 |
|  |  | MPDU1 |
|  |  | COQ6 |
|  |  | GLRX |
|  |  | MTHFS |
|  |  | RNASEH1 |
|  |  | OLR1 |
|  |  | DBT |
|  |  | TRHR |
|  |  | BEST1 |
|  |  | NDUFB10 |
|  |  | LTB4R |
|  |  | ALAS1 |
|  |  | EXO1 |
|  |  | RPS6KA6 |
|  |  | ATG7 |
|  |  | NOX4 |
|  |  | WIPF1 |
|  |  | USP24 |
|  |  | DNASE1 |
|  |  | CRHR2 |
|  |  | RNASE1 |
|  |  | ABCG8 |
|  |  | NFIB |
|  |  | EIF2S1 |
|  |  | KCNN3 |
|  |  | THPO |
|  |  | MB |
|  |  | BACH2 |
|  |  | APOD |
|  |  | RAD23A |
|  |  | P2RX3 |
|  |  | TRIM21 |
|  |  | PTPA |
|  |  | AP2B1 |
|  |  | DLG3 |
|  |  | NRG3 |
|  |  | U2AF1 |
|  |  | PLP1 |
|  |  | INHA |
|  |  | CALM3 |
|  |  | CD63 |
|  |  | PDGFA |
|  |  | DUOX1 |
|  |  | TACR2 |
|  |  | RAN |
|  |  | RNF216 |
|  |  | MCM6 |
|  |  | LRP4 |
|  |  | CUL1 |
|  |  | BAG3 |
|  |  | F11R |
|  |  | THY1 |
|  |  | HBEGF |
|  |  | CXADR |
|  |  | KCNQ4 |
|  |  | IL17F |
|  |  | ATP6V0A4 |
|  |  | PLEC |
|  |  | UCHL3 |
|  |  | GBE1 |
|  |  | TARS1 |
|  |  | POSTN |
|  |  | RASSF1 |
|  |  | OPRD1 |
|  |  | CYP2J2 |
|  |  | APH1A |
|  |  | TANK |
|  |  | GABARAP |
|  |  | EYA4 |
|  |  | IL9 |
|  |  | NRF1 |
|  |  | RICTOR |
|  |  | USP2 |
|  |  | KMT2D |
|  |  | NCOR2 |
|  |  | LZTR1 |
|  |  | EEA1 |
|  |  | GM2A |
|  |  | MOCS2 |
|  |  | FABP1 |
|  |  | ZDHHC9 |
|  |  | USF1 |
|  |  | FGF19 |
|  |  | SLC4A2 |
|  |  | TNFRSF25 |
|  |  | FADS1 |
|  |  | PIGA |
|  |  | TRPV3 |
|  |  | RCAN1 |
|  |  | ALCAM |
|  |  | NFATC3 |
|  |  | RPS6 |
|  |  | CHRND |
|  |  | MSH3 |
|  |  | CD2AP |
|  |  | SEL1L |
|  |  | SLC30A10 |
|  |  | TNR |
|  |  | PREX1 |
|  |  | COPA |
|  |  | RPS3 |
|  |  | CHL1 |
|  |  | HNRNPDL |
|  |  | CACNA2D3 |
|  |  | ESRRG |
|  |  | FYB1 |
|  |  | SLC14A2 |
|  |  | TAB1 |
|  |  | CCL20 |
|  |  | RUNX3 |
|  |  | RRAS |
|  |  | SGCG |
|  |  | TMEM43 |
|  |  | CITED2 |
|  |  | NADSYN1 |
|  |  | NPHP1 |
|  |  | ATP6V0A1 |
|  |  | CRTAP |
|  |  | ITGA9 |
|  |  | DLG1 |
|  |  | KPNA3 |
|  |  | GNB2 |
|  |  | KRT16 |
|  |  | LILRB1 |
|  |  | NSD2 |
|  |  | LTB4R2 |
|  |  | HIRA |
|  |  | RPS20 |
|  |  | HNF1B |
|  |  | HNRNPD |
|  |  | TCP1 |
|  |  | BAMBI |
|  |  | MLX |
|  |  | NEUROD1 |
|  |  | PLXNA2 |
|  |  | PYGB |
|  |  | CUL4A |
|  |  | PICK1 |
|  |  | PNPLA6 |
|  |  | SFPQ |
|  |  | TAC3 |
|  |  | ANLN |
|  |  | C3AR1 |
|  |  | NCK1 |
|  |  | NOD1 |
|  |  | TGM3 |
|  |  | POU2F1 |
|  |  | UTS2R |
|  |  | GRK5 |
|  |  | DNM3 |
|  |  | FKBP10 |
|  |  | PPP2R5C |
|  |  | ST3GAL4 |
|  |  | UGCG |
|  |  | AREG |
|  |  | CHST6 |
|  |  | FCN2 |
|  |  | TCN2 |
|  |  | TPSAB1 |
|  |  | YARS1 |
|  |  | GP1BB |
|  |  | BCOR |
|  |  | FBLN2 |
|  |  | KDM5A |
|  |  | NEFM |
|  |  | NUDC |
|  |  | TWIST2 |
|  |  | XRCC4 |
|  |  | EIF4A2 |
|  |  | FZD8 |
|  |  | HEY1 |
|  |  | LOXL1 |
|  |  | LRP8 |
|  |  | NUMA1 |
|  |  | PPP3CC |
|  |  | RGS10 |
|  |  | RNMT |
|  |  | RYK |
|  |  | SELENBP1 |
|  |  | SULT1A1 |
|  |  | WNT7B |
|  |  | FOXM1 |
|  |  | DST |
|  |  | PSMD2 |
|  |  | DGKB |
|  |  | FANCI |
|  |  | SLC3A2 |
|  |  | HR |
|  |  | PEX14 |
|  |  | B3GAT1 |
|  |  | NISCH |
|  |  | RPL13 |
|  |  | DSC3 |
|  |  | RPS26 |
|  |  | RACK1 |
|  |  | ANP32A |
|  |  | CDC37 |
|  |  | DNAJB2 |
|  |  | GNB4 |
|  |  | LIPF |
|  |  | TOP3A |
|  |  | DEPDC5 |
|  |  | HLA-DQB1 |
|  |  | EPO |
|  |  | ITIH4 |
|  |  | PYY |
|  |  | RPS27A |
|  |  | LRPPRC |
|  |  | ATP12A |
|  |  | TDGF1 |
|  |  | LTA |
|  |  | BST1 |
|  |  | PIGQ |
|  |  | GAS1 |
|  |  | VPS13A |
|  |  | VPS35 |
|  |  | PNKD |
|  |  | MPST |
|  |  | ATXN10 |
|  |  | KIF5C |
|  |  | PUF60 |
|  |  | AKAP9 |
|  |  | CNOT1 |
|  |  | FLG |
|  |  | SUMF1 |
|  |  | ADGRV1 |
|  |  | MCHR1 |
|  |  | CYP7A1 |
|  |  | CALB1 |
|  |  | GSTM1 |
|  |  | NPHS2 |
|  |  | LMX1A |
|  |  | DNAJC5 |
|  |  | JPH3 |
|  |  | HBA1 |
|  |  | ALDH9A1 |
|  |  | IFI16 |
|  |  | DMGDH |
|  |  | SLC10A2 |
|  |  | SBF1 |
|  |  | IGBP1 |
|  |  | HSPE1 |
|  |  | DTNBP1 |
|  |  | DARS2 |
|  |  | SETX |
|  |  | HECW2 |
|  |  | BANF1 |
|  |  | VARS2 |
|  |  | ASAH2 |
|  |  | OSGEP |
|  |  | CD69 |
|  |  | RAB11B |
|  |  | SMAD5 |
|  |  | DOCK7 |
|  |  | SDC3 |
|  |  | SPTBN2 |
|  |  | DHTKD1 |
|  |  | NDUFA8 |
|  |  | HAGH |
|  |  | GLE1 |
|  |  | RGS4 |
|  |  | TCAP |
|  |  | EIF4G3 |
|  |  | FKBP8 |
|  |  | HTR6 |
|  |  | SUCLG2 |
|  |  | HPGDS |
|  |  | AASS |
|  |  | PABPC1 |
|  |  | MMADHC |
|  |  | IYD |
|  |  | NBEA |
|  |  | COX15 |
|  |  | MGAT5 |
|  |  | CDH4 |
|  |  | PRKRA |
|  |  | GRIN3B |
|  |  | POLR2C |
|  |  | BCL11B |
|  |  | RREB1 |
|  |  | IRF2 |
|  |  | TIA1 |
|  |  | PAX7 |
|  |  | SCAP |
|  |  | FREM1 |
|  |  | PREP |
|  |  | AOC1 |
|  |  | DSG1 |
|  |  | APLP2 |
|  |  | PACS1 |
|  |  | ARHGAP4 |
|  |  | CTSE |
|  |  | PPBP |
|  |  | VAPA |
|  |  | STAG1 |
|  |  | SGCA |
|  |  | PSMD7 |
|  |  | SLC4A7 |
|  |  | GPAA1 |
|  |  | ST8SIA2 |
|  |  | NLGN2 |
|  |  | CRB2 |
|  |  | TREM1 |
|  |  | FMOD |
|  |  | CDH17 |
|  |  | TIMM8A |
|  |  | NDE1 |
|  |  | GPC5 |
|  |  | LIPG |
|  |  | SNAP91 |
|  |  | PNPLA3 |
|  |  | RAMP1 |
|  |  | PCSK6 |
|  |  | UBIAD1 |
|  |  | MACF1 |
|  |  | PSMD12 |
|  |  | ACD |
|  |  | IVNS1ABP |
|  |  | PIGB |
|  |  | FGF13 |
|  |  | PLA2G2D |
|  |  | EPB41L3 |
|  |  | GTF2H1 |
|  |  | COCH |
|  |  | SLC30A8 |
|  |  | IL22 |
|  |  | SRR |
|  |  | CELSR2 |
|  |  | CLIC1 |
|  |  | FABP5 |
|  |  | MLLT10 |
|  |  | RGMA |
|  |  | SLC44A2 |
|  |  | RCOR1 |
|  |  | ANAPC1 |
|  |  | ACSL3 |
|  |  | DGCR8 |
|  |  | UBQLN1 |
|  |  | CD276 |
|  |  | VPS37A |
|  |  | TLN1 |
|  |  | ANXA6 |
|  |  | SCNN1D |
|  |  | TREX1 |
|  |  | EFTUD2 |
|  |  | CHIA |
|  |  | PROCR |
|  |  | SLC35A3 |
|  |  | INPP1 |
|  |  | KDSR |
|  |  | PHKA1 |
|  |  | SH3KBP1 |
|  |  | CACNG1 |
|  |  | CBX3 |
|  |  | FTSJ1 |
|  |  | SLC4A5 |
|  |  | CERS2 |
|  |  | CPA3 |
|  |  | LAIR1 |
|  |  | PARVA |
|  |  | CCS |
|  |  | DDAH2 |
|  |  | NMBR |
|  |  | WDR26 |
|  |  | SULF1 |
|  |  | DDX11 |
|  |  | MAGT1 |
|  |  | MYPN |
|  |  | NUCB1 |
|  |  | RGS2 |
|  |  | SP7 |
|  |  | GIT1 |
|  |  | SMARCD1 |
|  |  | ARHGAP1 |
|  |  | RPL3 |
|  |  | GHRL |
|  |  | BMAL1 |
|  |  | TAC1 |
|  |  | TNFSF4 |
|  |  | GPT |
|  |  | NDN |
|  |  | PER1 |
|  |  | NALCN |
|  |  | POGZ |
|  |  | FLCN |
|  |  | GCG |
|  |  | PRRT2 |
|  |  | DHX30 |
|  |  | MEIS1 |
|  |  | ATP4A |
|  |  | PDSS2 |
|  |  | SLC25A22 |
|  |  | SLITRK1 |
|  |  | SLC37A4 |
|  |  | SRPX2 |
|  |  | AFF4 |
|  |  | GPR50 |
|  |  | IL16 |
|  |  | ATG4C |
|  |  | CCK |
|  |  | FOXH1 |
|  |  | PCDH15 |
|  |  | HAPLN1 |
|  |  | RAPSN |
|  |  | OTOF |
|  |  | HOMER1 |
|  |  | NAT2 |
|  |  | BAZ1B |
|  |  | LARP7 |
|  |  | DNAJB6 |
|  |  | ACKR1 |
|  |  | TRH |
|  |  | ATXN7 |
|  |  | ALMS1 |
|  |  | PECAM1 |
|  |  | NTS |
|  |  | SH3BP2 |
|  |  | GTF2IRD1 |
|  |  | GNE |
|  |  | FBXO11 |
|  |  | NDP |
|  |  | CCR8 |
|  |  | ADCYAP1 |
|  |  | CISD2 |
|  |  | VPS4A |
|  |  | SHBG |
|  |  | ANK2 |
|  |  | TNPO3 |
|  |  | UCP3 |
|  |  | ICOSLG |
|  |  | NAGS |
|  |  | PPIG |
|  |  | NPSR1 |
|  |  | XK |
|  |  | TCOF1 |
|  |  | VPS53 |
|  |  | ATP8B1 |
|  |  | SHANK2 |
|  |  | DDX39B |
|  |  | MSRA |
|  |  | KDM4C |
|  |  | SLC52A3 |
|  |  | SNCG |
|  |  | CXCR5 |
|  |  | SERPINA7 |
|  |  | ADAMTSL2 |
|  |  | FCGR1A |
|  |  | FOXE1 |
|  |  | ATL1 |
|  |  | LMOD1 |
|  |  | NDUFAF2 |
|  |  | NUP93 |
|  |  | PHF21A |
|  |  | KRT7 |
|  |  | CPSF3 |
|  |  | LAG3 |
|  |  | SDCCAG8 |
|  |  | LARGE1 |
|  |  | CRHBP |
|  |  | APOA4 |
|  |  | KLRK1 |
|  |  | FBN2 |
|  |  | RAB3A |
|  |  | TBX1 |
|  |  | MDC1 |
|  |  | CNPY3 |
|  |  | HOXA1 |
|  |  | PARS2 |
|  |  | VPS13B |
|  |  | SNIP1 |
|  |  | DLGAP1 |
|  |  | PORCN |
|  |  | TMLHE |
|  |  | RGS6 |
|  |  | SND1 |
|  |  | MFN1 |
|  |  | MC3R |
|  |  | S100A8 |
|  |  | TNFRSF18 |
|  |  | MPV17 |
|  |  | SEMA5A |
|  |  | MACROH2A1 |
|  |  | TRAF7 |
|  |  | CXCL11 |
|  |  | CDKN3 |
|  |  | SLC28A2 |
|  |  | ECI2 |
|  |  | C4B |
|  |  | ERF |
|  |  | AUTS2 |
|  |  | NPPC |
|  |  | ALDH1L2 |
|  |  | SP100 |
|  |  | SECISBP2 |
|  |  | CLPX |
|  |  | SLC25A29 |
|  |  | RPL10A |
|  |  | PPA2 |
|  |  | APOM |
|  |  | CNGB3 |
|  |  | POU1F1 |
|  |  | ATP5PO |
|  |  | RHOT1 |
|  |  | DOCK4 |
|  |  | ACAA1 |
|  |  | FKBP14 |
|  |  | RHD |
|  |  | SSBP1 |
|  |  | SURF1 |
|  |  | BIRC6 |
|  |  | NFU1 |
|  |  | FOXRED1 |
|  |  | HIBADH |
|  |  | MTHFD1L |
|  |  | TOP1MT |
|  |  | GK2 |
|  |  | MIPEP |
|  |  | MRPL3 |
|  |  | NDUFAF1 |
|  |  | MAGED1 |
|  |  | DDIT4 |
|  |  | ARHGEF9 |
|  |  | MRPS22 |
|  |  | KLK7 |
|  |  | NUP153 |
|  |  | BCL2A1 |
|  |  | CHM |
|  |  | CDX2 |
|  |  | HLCS |
|  |  | PPM1K |
|  |  | IFT81 |
|  |  | JMJD1C |
|  |  | PLAGL1 |
|  |  | CNTN4 |
|  |  | MAP1LC3B |
|  |  | KLK5 |
|  |  | CYFIP2 |
|  |  | IL13RA1 |
|  |  | AP1S2 |
|  |  | ADIPOR2 |
|  |  | S100A6 |
|  |  | TNPO1 |
|  |  | ZBTB17 |
|  |  | SPINK1 |
|  |  | ATP6V1H |
|  |  | HOXD13 |
|  |  | SIGLEC8 |
|  |  | PSMD9 |
|  |  | SH3GL2 |
|  |  | NR2E3 |
|  |  | SRCAP |
|  |  | IGSF1 |
|  |  | HNRNPH1 |
|  |  | HCCS |
|  |  | ZMPSTE24 |
|  |  | LGALS3BP |
|  |  | MUC5B |
|  |  | MED1 |
|  |  | TNFSF14 |
|  |  | SLC35A2 |
|  |  | CSGALNACT1 |
|  |  | GABARAPL2 |
|  |  | PMS1 |
|  |  | RAD18 |
|  |  | CENPJ |
|  |  | MBD1 |
|  |  | OSTM1 |
|  |  | RYR3 |
|  |  | RAB10 |
|  |  | NR0B2 |
|  |  | KCNV2 |
|  |  | HPSE2 |
|  |  | USP11 |
|  |  | GPX3 |
|  |  | NUP210 |
|  |  | RPGR |
|  |  | UBE3C |
|  |  | TET3 |
|  |  | FGD1 |
|  |  | IGHMBP2 |
|  |  | FAF1 |
|  |  | HIP1R |
|  |  | RAB1A |
|  |  | HNRNPC |
|  |  | HBG2 |
|  |  | UBE2E3 |
|  |  | LMO1 |
|  |  | FGFBP1 |
|  |  | CTNNA3 |
|  |  | MYO3B |
|  |  | COPS5 |
|  |  | OTUB1 |
|  |  | ADAMTS12 |
|  |  | DGKQ |
|  |  | UBE2S |
|  |  | SHOC2 |
|  |  | GCLM |
|  |  | CHGB |
|  |  | LDB3 |
|  |  | GPR55 |
|  |  | PPM1G |
|  |  | SLC45A2 |
|  |  | PDIA4 |
|  |  | SPEN |
|  |  | RAB6A |
|  |  | DNAJC3 |
|  |  | MT2A |
|  |  | BCL3 |
|  |  | SLC6A14 |
|  |  | DYNC1I2 |
|  |  | ACTR3 |
|  |  | AMFR |
|  |  | WASL |
|  |  | WWC1 |
|  |  | IL1RL1 |
|  |  | CC2D1A |
|  |  | CRELD1 |
|  |  | CLCN4 |
|  |  | SEC24B |
|  |  | SIX6 |
|  |  | HPX |
|  |  | KRIT1 |
|  |  | CDH6 |
|  |  | PABPC4 |
|  |  | MYO3A |
|  |  | ASAP1 |
|  |  | EEF1B2 |
|  |  | GCKR |
|  |  | MELTF |
|  |  | INHBB |
|  |  | SYN3 |
|  |  | SSB |
|  |  | CDSN |
|  |  | BMP15 |
|  |  | RAPGEF2 |
|  |  | HBA2 |
|  |  | KATNB1 |
|  |  | GMDS |
|  |  | KLRC1 |
|  |  | CA3 |
|  |  | TTPA |
|  |  | E2F3 |
|  |  | KMT2E |
|  |  | NUP133 |
|  |  | NUP85 |
|  |  | RAB3GAP1 |
|  |  | RTN2 |
|  |  | ALG3 |
|  |  | AP3D1 |
|  |  | CLP1 |
|  |  | F2RL2 |
|  |  | KLB |
|  |  | PAPPA2 |
|  |  | CETN2 |
|  |  | SIGLEC7 |
|  |  | YBX3 |
|  |  | HNRNPR |
|  |  | TIMP2 |
|  |  | PRX |
|  |  | ADARB2 |
|  |  | BRD1 |
|  |  | HOXA11 |
|  |  | KEL |
|  |  | RAB2A |
|  |  | RANBP9 |
|  |  | RECQL4 |
|  |  | RIMS1 |
|  |  | RPL7A |
|  |  | SOX11 |
|  |  | DNMBP |
|  |  | PCYT1B |
|  |  | KIF14 |
|  |  | NFAT5 |
|  |  | PDCD10 |
|  |  | S100A2 |
|  |  | AHI1 |
|  |  | CDH10 |
|  |  | COL10A1 |
|  |  | CRMP1 |
|  |  | EIF4B |
|  |  | IDH3G |
|  |  | ITSN1 |
|  |  | NAGK |
|  |  | HPS1 |
|  |  | ANKRD1 |
|  |  | ADD2 |
|  |  | AKAP12 |
|  |  | CHD1L |
|  |  | CHRD |
|  |  | CNNM2 |
|  |  | GMPPA |
|  |  | MAFA |
|  |  | PAWR |
|  |  | PAX4 |
|  |  | PCM1 |
|  |  | PIP4K2B |
|  |  | PLIN3 |
|  |  | PPP1R9B |
|  |  | QKI |
|  |  | RANGAP1 |
|  |  | RNF8 |
|  |  | RUNX1T1 |
|  |  | SARM1 |
|  |  | SERPINE2 |
|  |  | SNAP29 |
|  |  | SUZ12 |
|  |  | TFAP2C |
|  |  | WWP1 |
|  |  | IRS4 |
|  |  | ECT2 |
|  |  | NMNAT2 |
|  |  | SPTB |
|  |  | MCC |
|  |  | AATF |
|  |  | AIM2 |
|  |  | LRIG2 |
|  |  | SERPINA4 |
|  |  | SORBS1 |
|  |  | BACH1 |
|  |  | TNFRSF12A |
|  |  | CLASP1 |
|  |  | LTBR |
|  |  | PTGER1 |
|  |  | TRIM2 |
